# comBO: A combined human bone and lympho-myeloid bone marrow organoid for preclinical modeling of hematopoietic disorders

**DOI:** 10.1016/j.stem.2026.01.010

**Published:** 2026-02-23

**Authors:** Yuqi Shen, Camelia Benlabiod, Edmund Watson, Kristian Gurashi, Alex Fower, Antonio Rodriguez-Romera, Jasmeet S. Reyat, Shady Adnan-Awad, Rupen Hargreaves, Samuel Kemble, Charlotte G. Smith, Adam P. Croft, Udo Oppermann, Alia Welsh, Lauren Murphy, Eleanor Murphy, Amirpasha Moetazedian, Natalie Jooss, Zoe C. Wong, Julie Rayes, Adam J. Mead, Anindita Roy, Sarah Gooding, Bethan Psaila, Abdullah O. Khan

**Affiliations:** 1 MRC Molecular Haematology Unit, Weatherall Institute of Molecular Medicine, Radcliffe Department of Medicine and National Institute of Health Research (NIHR), Oxford Biomedical Research Centre, University of Oxford, Oxford, UK; 2 Oxford Translational Myeloma Centre, Botnar Research Centre, University of Oxford, Oxford, UK; 3 Division of Cardiovascular Medicine, Radcliffe Department of Medicine, University of Oxford, John Radcliffe Hospital, Oxford, UK; 4 Rheumatology Research Group, Department of Inflammation and Ageing, College of Medicine and Health, University of Birmingham, Birmingham, UK; 5 National Institute for Health and Care Research (NIHR) Birmingham Biomedical Research Centre, Birmingham, UK; 6 School of Engineering and Technology, Faculty of Science and Engineering, Hull University, Hull, UK; 7 Department of Cardiovascular Sciences College of Medicine and Health, School of Medical Sciences, University of Birmingham, Vincent Drive, Birmingham, UK; 8 Department of Paediatrics, University of Oxford, Oxford, UK; 9 Ludwig Institute for Cancer Research, Nuffield Department of Medicine, University of Oxford, Oxford, UK

## Abstract

The bone marrow is the primary site of blood and immune cell production in postnatal life. Current human models do not capture lympho-myeloid hematopoiesis and the stromal diversity needed for lifelong blood and immune maintenance. Here, we introduce comBO (combined bone and lympho-myeloid bone marrow organoid), a scalable induced pluripotent stem cell (iPSC)-derived system that generates osteolineage, vascular, lymphoid, and myeloid compartments within a single organoid. Developed under physioxia in granular microgel scaffolds, comBOs improve scalability and reproducibility and sustain long-term lympho-myeloid potential in serial organoid re-seeding assays. Incorporating healthy or malignant donor cells produces “chimeroids” that model physiological and pathological states. Using multiple myeloma as an exemplar, comBOs recapitulate niche remodeling and identify macrophage inhibitory factor (MIF) signaling as a disease driver. MIF inhibition reduces inflammation and myeloma proliferation, highlighting its therapeutic potential. comBOs offer a physiologically faithful bone marrow platform for disease modeling and therapeutic discovery in translational hematology and immunology.

## Graphical abstract

**Graphical abstract:**
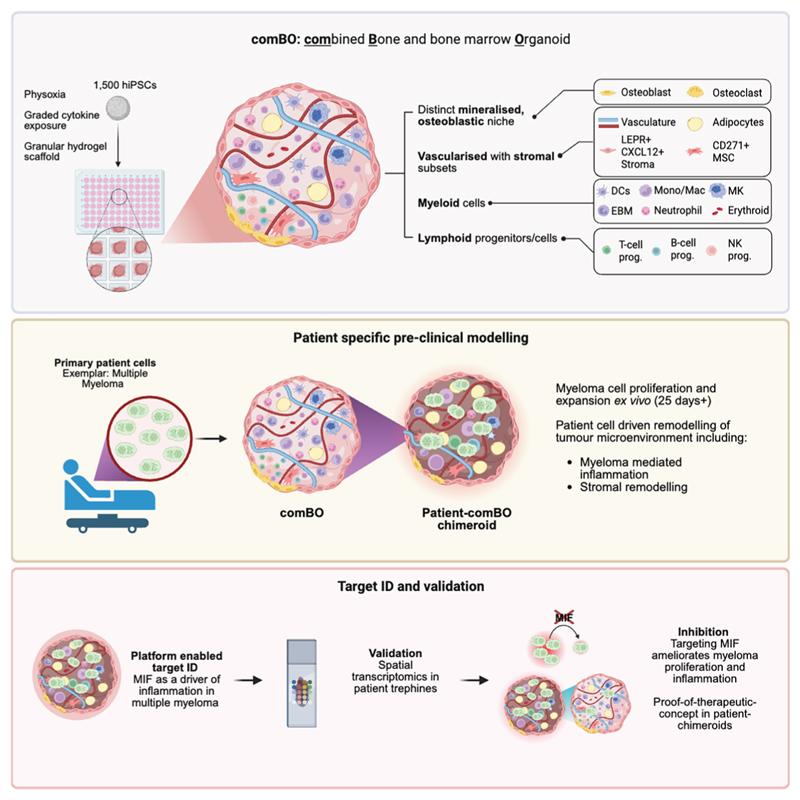

## Introduction

The bone marrow is the primary site of postnatal hematopoiesis and is organized into specialized niches that support blood and immune cell production.^1,2^ Vascular and stromal components regulate hematopoietic stem and progenitor cell (HSPC) differentiation, quiescence, and expansion.^3–5^ The stromal repertoire of the bone marrow includes mesenchymal stromal cells (MSCs), which generate osteo- and adipo-lineage cells, as well as specific MSC subsets (e.g., leptin receptor [LEPR+] and nestin [NES+] MSCs).^1,4,6^ These extrinsic cues maintain hematopoiesis at steady state and during physiological challenges.

Disruption of the bone marrow microenvironment contributes to the pathogenesis of bone marrow disorders, including blood malignancies and bone marrow failure syndromes.^7^ Disease-mediated changes in the microenvironment, such as inflammation, are known to limit therapeutic efficacy and contribute to chemoresistance and relapse.^7,8^ Similarly, age-associated changes to the niche contribute to the myeloid-biased hematopoiesis and immune decline that occurs during human ageing.^9^ However, human bone marrow is relatively inaccessible, and Species-specific differences limit the translatability of animal models.^10–12^ Human *in vitro* systems are therefore required for mechanistic and translational studies.

Previous approaches have implemented microfluidic^13–15^ and three-dimensional (3D) scaffolds^16–19^ to model human bone marrow *in vitro*. Typically, these strategies rely on primary cells, resulting in batch variability, reproducibility, and Scalability. Moreover, they have not supported long-term functional hematopoietic stem cell (HSC) maintenance or incorporated bone marrow-specific stromal diversity.

Recent advances in the development of human induced pluripotent stem cell (hiPSC)-derived bone marrow organoids have begun to address this gap.^17,19–22^ We, and others, have recently developed myeloid bone marrow organoids (mBMOs) composed of myelopoietic cells, endothelium, fibroblasts, and MSCs^23,24^ and demonstrated their utility in modeling myeloid disorders and genetic hematopathologies.^25^ Most recently, Li et al. report a model of endosteal niches (eVON [engineered vascularized osteoblastic niche]), using a hydroxyapatite scaffold to generate a vascularized model of the endosteum.^22^ Despite these advances, current models have three key limitations: they do not capture simultaneous myelopoiesis and lymphopoiesis, they lack both the functionally distinct adipocytic and osteoblastic niches, and their scalability and reproducibility are limited by the requirement for embedding and extraction from hydrogel scaffolds.

To address these limitations, we developed combined bone and lympho-myeloid bone marrow organoids (comBOs), a scalable, physiologically relevant multi-niche model of the human bone marrow. comBOs generate both lymphoid and myeloid lineage cells and contain a complexity of stromal cell types including osteolineage, adipocyte, and MSC subtypes with transcriptional homology to adult bone marrow. Use of granular microgel scaffolds greatly improves the scalability and reproducibility compared with bulk hydrogel-based approaches, enabling higher-throughput organoid production for discovery and preclinical applications.

We demonstrate the utility of comBOs to model human disease by engrafting cells from patients with multiple myeloma, a plasma cell (PC) malignancy characterized by inflammatory niche remodeling and bony destruction.^26,27^ Myeloma-engrafted comBO “chimeroids” faithfully capture stromal inflammation, metabolic reprogramming of MSCs, and arrested maturation of osteoblasts. Dissecting receptor-ligand interactions between myeloma cells and organoid niche components identified macrophage inhibitory factor (MIF) as a central mediator of myeloma-driven inflammation, and we show that inhibition of MIF mitigates the inflammatory responses induced by primary myeloma cells.

Together, comBOs represent a scalable, multi-niche human organoid model for mechanistic studies of bone marrow biology and translational investigation of disease.

## Results

### Development of comBO: A single differentiation generating lympho-myeloid, vascular, and osteogenic cell types

**Figure 1:**
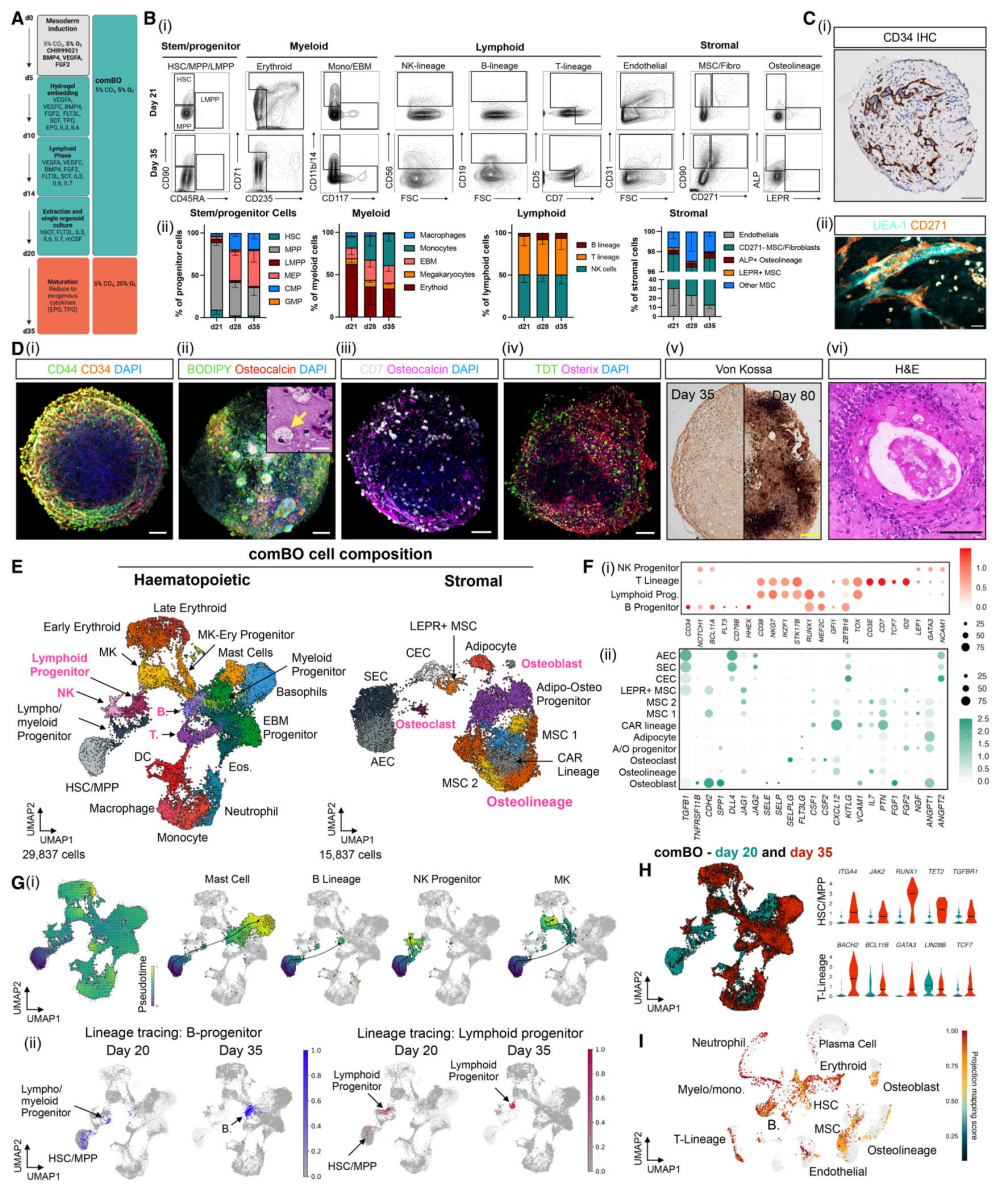
Development and validation of a protocol to generate a hiPSC-derived comBO (A) Schematic of workflow. comBOs are generated in a bone marrow-relevant oxygen concentration (5% O_2_). Schematic generated on Biorender.com. (B) (i) Representative flow plots of key comBO hematopoietic and stromal populations at day 21, and maturation of those populations by day 35. (ii) Quantified relative percentages of hematopoietic and stromal cell types in each flow panel over time (*n* = 4 separate differentiations, 24 organoids pooled per repeat). Each cell type was identified and quantified using the gating strategy shown in Figures S1B–S1E, followed by the calculation of its relative proportion within the defined populations. (Error bars represent standard deviation). (C) (i) Validation of lumen-forming vascular architecture using CD34 staining (scale bar, 100 μm) and (ii) confocal microscopy (vasculature [UEA-1], perivascular MSC [CD271+], scale bar, 25 μm). (D) Confocal imaging to confirm (i) vasculature, (ii) adipogenesis (BODIPY+ cells and highlighted by yellow arrow in H&E insert; scale bars, 100 μm and 20 μm, respectively) and early osteogenesis (osteocalcin+), and (iii and iv) lymphoid cells in proximity to osteolineage regions (CD7-osteocalcin and TDT-osterix) (scale bars, 100 μm). (v) Von Kossa staining (scale bar, 200 μm) and (vi) H&E staining (scale bar, 50 μm) to confirm bone formation by day 80. (E) Uniform manifold approximation and projection (UMAP) of single-cell data characterizing the hematopoietic and stromal cells generated at days 20 and 35 by comBOs to transcriptionally profile cells and assess maturation. MK, megakaryocytes; EBM, eosinophil/basophil/mast cells; DC, dendritic cells; CEC, capillary endothelial cells; AEC, arteriolar endothelial cells; SEC, sinusoidal endothelial cells; MSC, mesenchymal stem cells. (F) Bubble plots characterizing (i) gene expression in lymphoid populations, and (ii) the expression of key bone marrow stromal markers and factors. (G) (i) Lineage tracing using Palantir and CellRank (pseudotime measurements) to confirm HSC/MPP-derived lineage trajectories in key myeloid and lymphoid lineages. (ii) Application of Moscot method to confirm trajectories in key populations using temporally resolved data. (H) Comparison of days 20 and 35 comBO transcriptome highlighting canonical changes in the expression of adult genes in HSC/MPP and T-lineage. (I) Integration with a recently published atlas of adult human bone marrow revealing homology and overlap with cells of the adult bone marrow. See also Figures S1–S3.

To generate a 3D culture capturing lympho-myeloid and bone marrow stromal elements, human iPSCs were subjected to a series of cytokine cocktails and hydrogel embedding to guide multi-lineage differentiation (Figure 1A, detailed schematic: Figure S1A).^28^ Given that the bone marrow is a low-oxygen environment where hypoxia is essential for HSC regulation and stromal cell differentiation,^29–31^ cultures were maintained at 5% O_2_ for 20 days in contrast to the 72-h mesodermal induction used in prior studies.^23,24^ To promote and support lymphopoiesis, a lymphoid phase was introduced at day 10, in which myeloid-promoting cytokines were withdrawn and the lymphopoietic cytokine IL-7, a critical regulator of B and T cell development, was added (Figure S1A).^32^

This approach generated HSPCs, myeloid cells (erythroid, megakaryocyte, monocyte, and eosinophil/basophil/mast [EBM] cells), lymphoid cells (T-lineage progenitors and natural killer [NK] cells), mesenchymal stromal, osteolineage, and endothelial cells at day 21 of culture as demonstrated by flow cytometry (Figures 1B(i) and S1B–S1G; Table S1). A further two-week culture at 20% O_2_ allowed for the maturation of key populations, including CD19+ B cells, while T-lineage cells progressed from a CD7+CD5– phenotype at day 21 to CD5+CD7+ on day 35 of culture (Figures 1B, S1F, and S1G).

Imaging confirmed a lumen-forming, CD34+ vascular network (Figure 1C[i]), with vessels supported by CD271+ perivascular cells (Figure 1C[ii]). 3D confocal imaging was performed to confirm the architecture of comBO: CD34+ vasculature and hematopoietic cells (Figure 1D[i]), adipogenesis and early osteogenesis (BODIPY+ large cells, osteocalcin deposition; Figure 1D[ii]), the accumulation of lymphoid lineage cells in regions positive for early osteolineage and mineralization markers (CD7/osteocalcin and TDT [terminal deoxynucleotidyl transferase/Osterix, respectively]) (Figure 1D[iii and iv]). Finally, the functionality of osteolineage cells was confirmed by assessing the progressive mineralization of comBO over time using Von Kossa staining and alizarin red (Figures 1D[v] and S1H), confirming the presence of osteolineage cells with bone-depositing capacity. H&E staining confirmed the formation of early bone-like morphology as mineralization occurs (Figure 1D[vi]).

We confirmed the clonogenic potential of HSPCs by colony-forming unit (CFU) assay, using comBO-derived CD34+ cells compared with peripheral blood controls (Figures S1I and S1J). Together, these data demonstrate generation of lymphoid, myeloid, vascular, and mesenchymal stromal lineages, including functional osteogenesis and adipogenesis, within a single human organoid system.

### comBOs show transcriptional homology to adult human bone marrow

To investigate the homology of the organoid cell types to those present in human bone marrow, single-cell RNA sequencing (scRNA-seq) was performed on organoids at two time points (days 20 and 35). Together, cells from 4 independent differentiations were pooled to generate a dataset of 29,837 hematopoietic and 15,837 stromal cells (Figures 1E, S2A, and S2B). Cell types were annotated using canonical markers (Figures 1F, S2C, and S2D).^2,33,34^

The hematopoietic compartment included monocyte/macrophage cells, neutrophils, dendritic cells, lymphoid and myeloid progenitors, T cell progenitors, EBM cells, HSPCs, erythroid cells, and megakaryocytes (Figures 1E, 1F, S2C, and S2D). The stromal compartment was comprised of endothelial cells (sinusoidal [SEC], arterial [AEC], and capillary [CEC] endothelial cells), MSCs, CAR (CXCL12-abundant reticular) progenitors, adipocyte/osteoblast lineage cells, and fibroblasts (Figures 1E, 1F, S2C, and S2D).

comBO demonstrated, for the first time, the generation of lymphoid populations in parallel to myeloid cells in a single organoid differentiation.^23,25^ These included B cell progenitor (*CD34, CD79B, FLT3*, and *BCL11A*), lymphoid progenitor (*CD38, NKG7, IKZF1, RUNX1, MEF2C*, and *TOX*), T cell lineage (*CD3E, CD7*, and *TCF7*), and NK cell progenitor (*LEF1, GATA3*, and *NCAM1*) populations (Figures 1F[i] and S2C). Similarly, osteoblasts (*POSTN, SPP1, ANGPT1, KITLG, KLL1, WNT5A, SP7*, and *BGLAP*), osteolineage progenitors (*BMP2, ALPL, JAG1, GREM1*, and *CDH11*), and early osteoclasts (*MMP9, TNFRS11A*, and *TNFSF11*) were identified (Figure S2C). Stromal cells expressed key hematopoietic support factors including *KITLG, TGFB1, DLL4, JAG1/2, CXCL12, IL7*, and *ANGPT1/2*, confirming the production of hematopoietic support factors by the organoid niche components (Figure 1F[ii]).

Lineage trajectory analysis confirmed HSC/MPP (multipotent progenitor cell) differentiation into both myeloid and lymphoid lineages (confirmed by both Palantir pseudotime analysis and Moscot-based sampling informed by days 20 and 35 samples, Figure 1G[i and ii]).

A comparison of days 20 and 35 gene expression data revealed an increase in the expression of markers of adult hematopoiesis, indicating a maturation of comBO hematopoietic cells (Figures 1H, S2E, and S2F). HSC/MPPs showed a notable increase in *ITGA4, JAK2, RUNX1, TET2*, and *TGFBR1*, and T-lineage cells demonstrated an increase in *BACH2, BCL11B, GATA3*, and *TCF7* and a decrease in fetal *LIN28B* over time (Figure 1H).

Finally, day 35 data was integrated with a recently published adult human bone marrow atlas to reveal a high degree of transcriptional homology between comBO and adult bone marrow cells (Figures 1I and S3A–S3C).^2^ Compared with fetal bone marrow atlas data and recently published organoid models, comBO demonstrated consistently higher homology to adult data consistent with the trends toward adult-like gene expression observed (Figures S3A–S3C). A comparison of gene expression data from comBO with previously published bone marrow atlas data sets confirmed the breadth of cell types captured by comBO, as well as the expression of key bone marrow-specific stromal markers and growth factors (e.g., *LEPR, CXCL12, IL7, RUNX2*, and *NFATC1*) (Figure S3D).

### Using granular microgels enables high-throughput organoid generation

The use of “bulk” hydrogels creates limitations to scalability and cost due to labor-intensive preparation, the requirement for manual extraction, and replating of organoids, which preclude automation for higher-throughput pharmacogenomic studies.^23–25^

**Figure 2:**
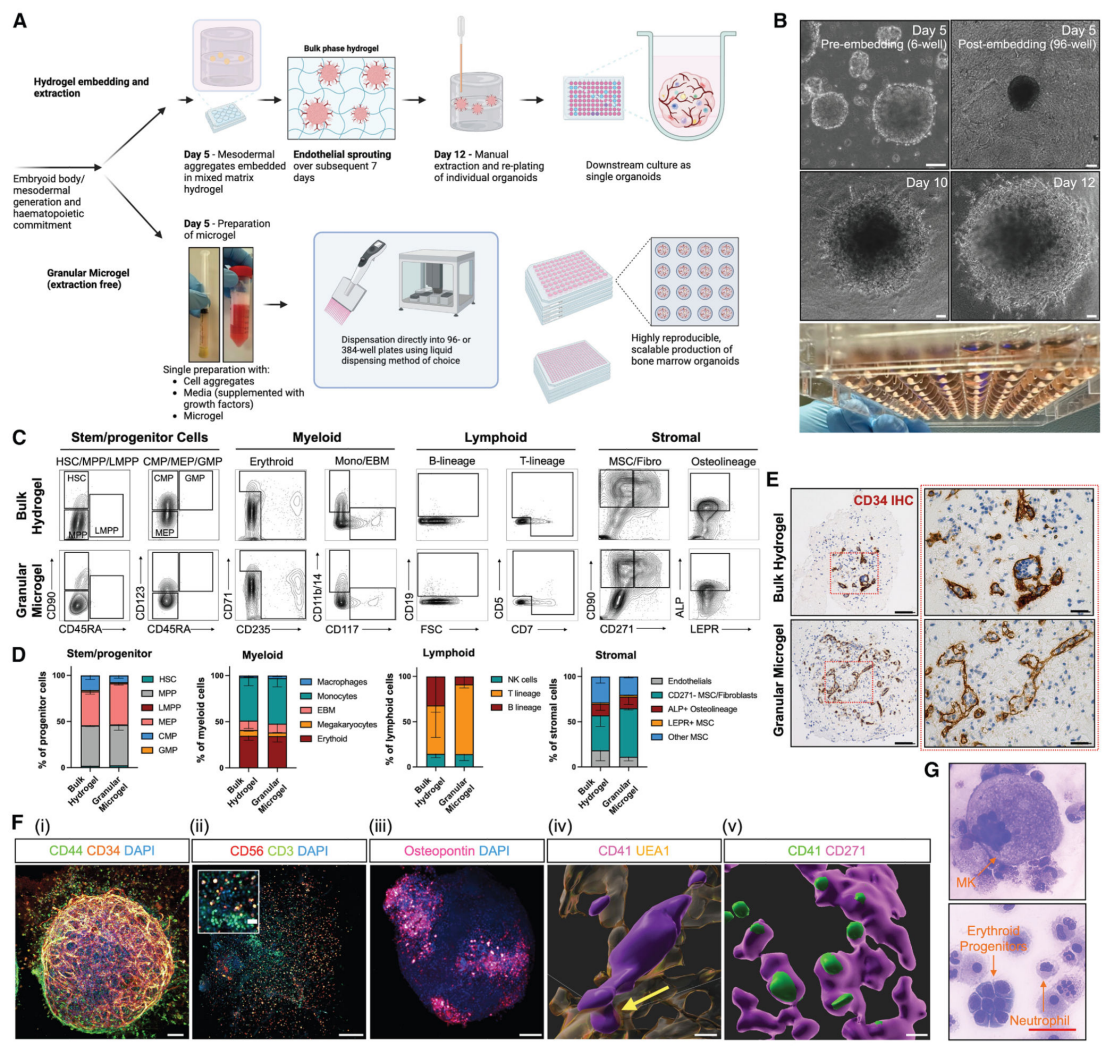
Development and validation of a high-throughput, granular microgel method of comBO generation (A) A granular microgel method was devised as an alternative to conventional bulk hydrogel approaches. Generated on Biorender.com. Step-by-step description of granular microgel protocol described in Figure S4B. (B) Mesodermal aggregates embedded in the granular microgel at day 5 develop vascular sprouts over the following 7 days and mature without the requirement for manual extraction (scale bar, 100 μm). (C) Flow cytometry at day 35 of differentiation confirms the equivalent generation of key stromal and hematopoietic lineages using the granular microgel approach. (D) Quantified relative proportions of hematopoietic and stromal cell types in each flow panel. (*n* = 3. Error bars are standard deviation). Gating strategy for flow cytometry in Figures S1B–S1E. Representative plots and quantification of comBOs generated from multiple hiPSC lines shown in Figures S4C and S4D. (E) Both protocols generate a lumen-forming vascular network as confirmed by CD34 immunohistochemistry (IHC) in paraffin-embedded sections (scale bar, 100 μm; insert, 25 μm). (F) Confocal imaging confirms (i) a CD34+ vascular network and structure invested with CD44+ cells (scale bar, 100 μm), (ii) lymphoid cells (CD56+ and CD3+ cells; scale bar, 200 μm), (iii) osteopontin deposition (scale bar, 200 μm), (iv) CD41+ MKs producing proplatelet extensions into vascular lumen (scale bar, 5 μm), and (v) associating with CD271+ MSCs (scale bar, 20 μm). (G) Cytospins of dissociated comBOs reveal a complex mix of morphologies (scale bar, 50 μm), annotated in detail in Figure S4E. See also Figure S4.

Inspired by granular microgel approaches, we developed a workflow to overcome the current limitations of bulk hydrogel embedding.^35–38^ Hydrogels were mechanically processed to generate a granular microgel (Figures 2A, S4A, and S4B), then combined with media, growth factors, and cell aggregates and dispensed into wells of 96- (or 384-) well ultra-low attachment plates followed by centrifugation to compact the microgel (Figure 2A).^36^ We found that the compacted microgels supported vascular sprouting in a similar manner to bulk hydrogels while using a fraction of the material (80% reduction in bulk gel volume) (Figure 2B). This approach eliminates the need for manual extraction during culture.

A direct comparison of parallel differentiations performed to generate comBOs using bulk hydrogel and granular microgel methods showed no loss of cell populations or architectural complexity using the microgel approach (Figures 2C–2E). Flow cytometry confirmed the generation of myeloid, lymphoid, and Stromal lineages across three independent hiPSC lines (Figures 2C, 2D, S4C, and S4D). Imaging further verified the formation of lumen-forming vessels (Figure 2E), similar to the architecture of organoids generated using bulk hydrogels. Confocal imaging confirmed an elaborate 3D vasculature (Figure 2F[i]), lymphoid (CD3+ CD56+) cells (Figure 2F[ii]), osteopontin-rich regions (Figure 2F[iii]), and megakaryocytes extending proplatelet protrusions into the vascular space (highlighted by a yellow arrow, Figure 2F[iv]), and closely associating with CD271+ MSCs (Figure 2F[v]).

Cytospins of comBO cells derived from the granular microgels highlight a range of morphologies consistent with mature myeloid cells (megakaryocytes, neutrophils erythroid progenitor clusters, and macrophages) and lymphoid progenitors (Figures 2G and S4E). Thus, granular microgels generate comBOs of equivalent complexity while enabling scalable production.

### Functional assessment of comBO-derived cells

**Figure 3:**
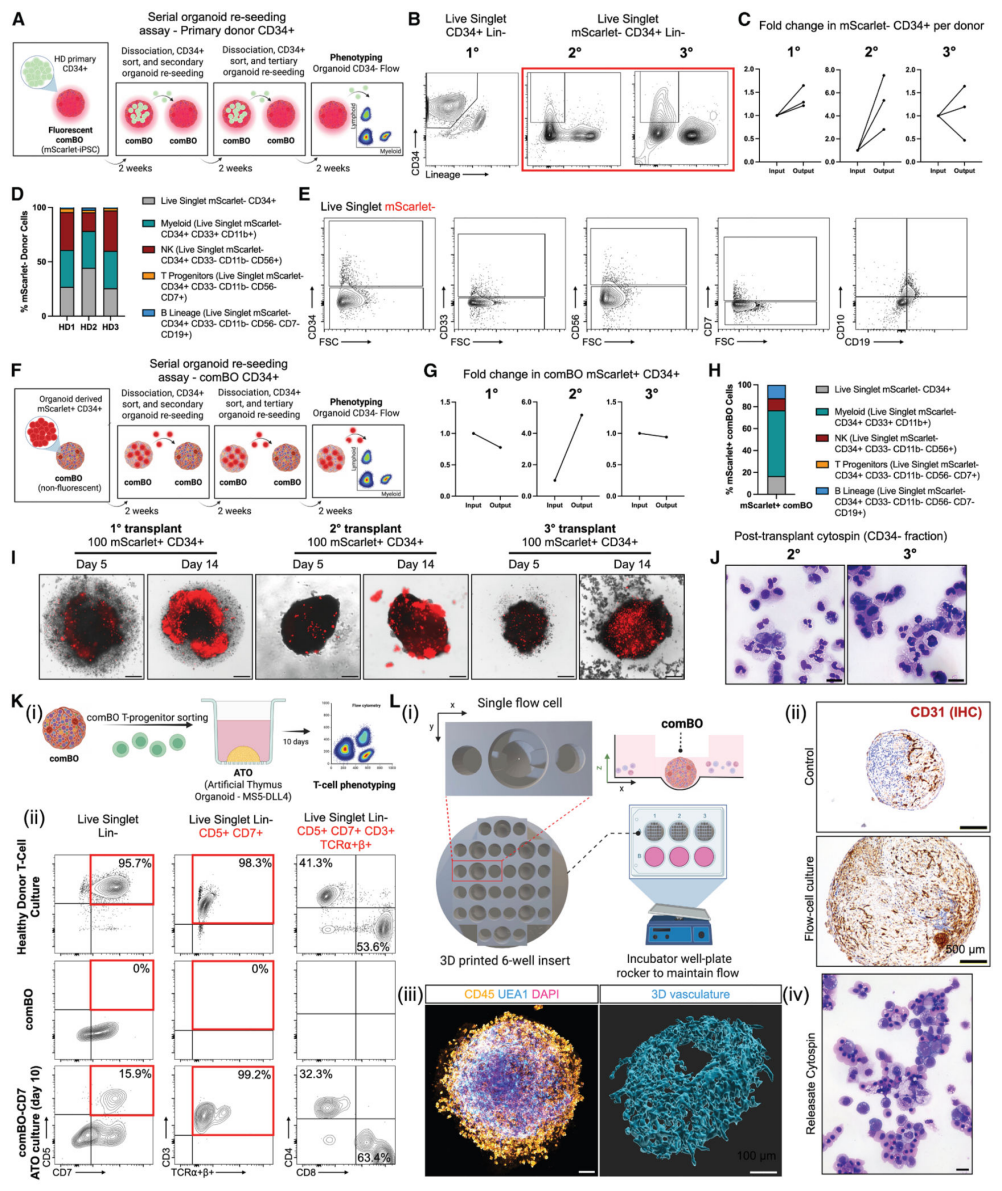
Functional validation of comBO-derived cells (A) Workflow for the serial re-seeding of primary human CD34+ cells in comBO. (B) Representative plots of CD34+ cells at each serial re-seed and cell sorting time point confirming CD34 expression. (C) Quantification of fold change in the number donor CD34+ cells at each re-seeding time point. (D) Quantification of percentage of lymphoid, myeloid, and CD34+ cells post re-seeding, confirming lympho-myeloid potential post transfer. (E) Representative plots of key lineages after re-seeding (gated on mScarlet-donor cells). (F) Workflow for the serial re-seeding of mScarlet CRISPR-knockin comBO-derived CD34s into non-fluorescent comBOs. (G) Fold change in comBO mScarlet+ CD34+ cells over 3 serial organoid re-seeds. (H) Quantification of CD34+ expression and major lymphoid and myeloid lineages after re-seeding. (I) Imaging of mScarlet CD34+ cells at day 5 and day 14 of each re-seeding cycle, demonstrating the expansion of CD34+ cells after sorting and engraftment. (J) Cytospin of CD34− cells following serial re-seeding confirming the morphology of CD45+ cells. Scale bar, 10 μm. (K) Validation of maturation potential of comBO-derived CD7+ T-lineage progenitors after engraftment and maturation in an artificial thymus organoid (ATO) system. (L) Validation of comBO vasculature development under dynamic flow. (i) Schematic of 3D printed flow cell device. (ii) Histology of control (static well-culture) and comBO cultured under flow for 10 days confirming the elaboration of the organoid vasculature and size under flow (CD31 IHC; scale bar, 500 μm). (iii) Confocal imaging of CD45+ cells accumulating at the periphery of comBO under flow and an elaborate 3D vasculature. (iv) Cytospin of cells released by comBO under flow confirming hematological morphology. Scale bar, 10 μm.

To test whether comBO can mimic bone marrow function in maintaining HSCs long term, we established a comBO-based serial organoid re-seeding assay (Figure 3A). Primary CD34+ cells from three healthy donors were seeded into mScarlet-tagged (CRISPR knockin) hiPSC-derived comBOs at a density of 100 CD34+ cells per organoid and cultured for 2 weeks in growth factor-free media. After 2 weeks, comBOs were dissociated and CD34+ mScarlet– cells were re-seeded at the same density (secondary re-seeding). A further passage was performed after two additional weeks (tertiary re-seeding). At week 8, following three successive seedings, the lympho-myeloid potential of donor cells was assessed.

All three donor samples retained a CD34+ Lineage– fraction (Figure 3B) at each seeding stage, demonstrating a consistent fold increase in CD34+ cells as well as lineage+ differentiation across engraftments (Figures 3B and 3C). All 3 donors demonstrated an increase in CD34+ cell number after the first (HD1 1.26×-, HD2 1.21×-, and HD3 1.64×-fold) and second (HD1 2.82×-, HD2 5.35×-, and HD3 7.52×-fold) seedings. 2 donors demonstrated a continued expansion after tertiary re-seeding (HD1 1.19×- and HD2 5.35×-fold increase), while the third showed a decline (HD3 0.49×-fold decrease) in CD34+ cell number. Flow cytometry confirmed that post re-seeding, donor cells retained lympho-myeloid potential (Figures 3D and 3E), confirming that comBO can sustain CD34 expression and lymphomyeloid potential long term without the supplementation of exogenous cytokines.

We next asked whether comBO-derived CD34+ cells themselves could retain their HSPC activity through a long-term, serial re-seeding assay (Figure 3F). Using mScarlet-labeled hiPSCs, we generated comBO CD34+ cells and seeded them into non-fluorescent comBOs. As with primary donor cells, hiPSC-derived CD34+ cells were maintained across three serial re-seedings and retained lympho-myeloid potential under growth factor-free conditions over 8 weeks (Figures 3G–3J).

To test whether comBO-derived T cell progenitors can differentiate into mature T cells, we engrafted comBO CD7+ cells into an artificial thymus organoid (ATO) (Figure 3K[i]). Ten days after engraftment, we detected expression of CD3 and TCRα/β, as well as the emergence of CD4+ and CD8+ single-positive cells (Figure 3K[ii], flow panel in Table S1). This confirms that comBO-derived T cell progenitors can generate mature T cells.

Finally, we evaluated the capacity of the comBO vasculature to develop under dynamic flow by seeding comBOs into 3D-printed fluidic devices (Figure 3L[i]). Under flow, comBOs developed a more elaborate vascular network that facilitated the egress of hematopoietic cells (Figure 3L[ii–iv]), confirming that the comBO vasculature can respond to physiological flow cues.

### comBOs support primary multiple myeloma cells *ex vivo* and capture multilineage pathogenesis and remodeling

Having established that comBO captures key features and functions of the human bone marrow niche, we next explored its utility to recapitulate tumor-microenvironment interactions. Multiple myeloma is a PC malignancy in which relapse remains a major clinical challenge, accompanied by extensive remodeling of the bone and marrow niche. In patients, osteolineage, endothelial, adipogenic, immune, and myeloid compartments are disrupted and contribute to disease progression and relapse.^26,39–43^ By integrating stromal and hematopoietic elements, comBO enables analysis of tumor-microenvironment interactions.

**Figure 4:**
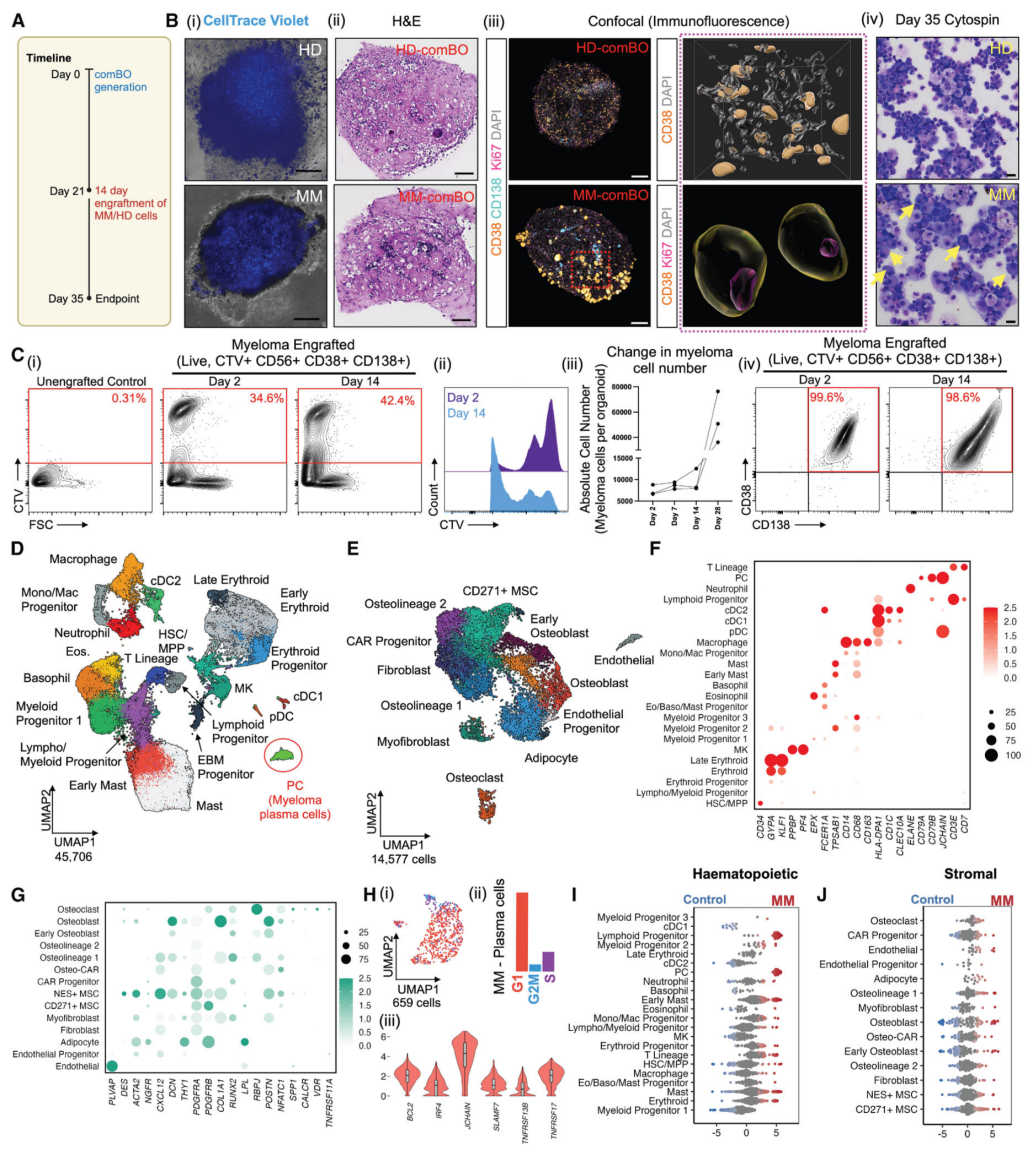
comBOs support the engraftment of primary multiple myeloma cells (A) CD138+ bone marrow cells from patients with myeloma (*n* = 3) and CD34+ healthy donor (HD) cells (*n* = 1) were labeled with CellTrace Violet and engrafted into comBOs at day 21 of differentiation and cultured for a further 14 days. Schematic created on Biorender.com. (B) (i) Imaging of engrafted samples reveals CellTrace^+^ cells successfully engraft comBOs (scale bar, 600 μm). Full details shown in Figures S5A and S5B. (ii) Paraffin-embedded histological sections of control and MM-comBO (scale bar, 100 μm). (iii) Confocal imaging confirmed CD38+ CD138+ large myeloma cells in engrafted samples within the 3D volume, which co-stained for Ki67 confirming cell proliferation. (iv) Dissociated samples at day 14 post engraftment (day 35 of protocol) show viable myeloma cells (scale bar, 25 μm). (C) (i and ii) Flow cytometry confirmed the proliferation of myeloma cells as shown by cell trace dilution over time. (iii) Quantification of the absolute number of myeloma cells over time shows an increase in cell number across all 3 donors tested. (iv) Immunophenotyping of myeloma cells post engraftment confirms CD38+ CD138+ and CD56+ expression. (D) Hematopoietic cells (45,706) of the integrated myeloma, HD, and control un-engrafted samples with the patient specific plasma cells (PCs) circled. (E) Stromal cells (14,577) from the same samples. (F and G) Simplified representative bubble plots highlighting key hematopoietic and stromal lineage markers. Full list shown in Figures S5C and S5D. (H) Myeloma plasma cells (PCs) were sub-clustered and scored using Seurat’s cell-cycle scoring method (i and ii). (iii) Cells expressing hallmark myeloma genes (*BCL2, IRF4, JCHAIN, SLAMF7, TNFRSF13B*, and *TNFRSF17*) were observed. (I and J) (I) Differential abundance (DA, MiloR) analysis of hematopoietic and (J) stromal cells highlight myeloma-induced changes in cell abundance. See also Figure S5.

We engrafted comBOs with CD138+ cells from cryopreserved bone marrow aspirates from three patients with high-risk multiple myeloma to generate myeloma-comBO chimeroids (MM-comBO, Figure 4A; Table S2). Chimeroids were cultured for 2 weeks before flow cytometry, scRNA-seq, and imaging. As controls, un-engrafted organoids and organoids engrafted with CD34+ cells from the peripheral blood of a healthy granulocyte-colony stimulating facor (G-CSF)-mobilized adult donor were processed under identical conditions (Figures 4A, S5A, and S5B).

Healthy donor CD34+ and myeloma cells were labeled with CellTrace Violet on day 0 prior to engraftment to enable tracking. Organoids were then imaged on days 3, 7, and 14 (Figures 4B[i] and S5B). Engrafted cells were detected within organoids from day 3 onward. Internalization of the myeloma cells was confirmed by histology (H&E) (Figure 4B[ii]) and confocal microscopy (Figure 4B[iii], which demonstrated CD38+CD138+ myeloma cells in MM-comBOs co-expressing the proliferation marker Ki67 (Figure 4B[iii]). Cytospins of dissociated chimeroids confirmed the presence of PCs (Figure 4B[iv]), which were further analyzed using flow cytometry (Table S1). Flow analysis of the engrafted organoids at days 2 and 14 further confirmed the presence and expansion of proliferating CellTrace Violet+ cells (Figure 4C[i–iii]), which retained CD38, CD56, and CD138 expression after 2 weeks (Figure 4C[iv]). Total myeloma cell number was assessed at an additional time point to demonstrated a continued expansion of myeloma cells at day 28 post engraftment under minimal cytokine supplementation (5 ng/mL erythro-poietin [EPO] and 1 ng/mL thrombopoietin [TPO]) (Figure 4C[iii]).

scRNA-seq data was analyzed and clusters were annotated using canonical marker genes (Figures 4D–4G, S5C, and S5D) to identify hematopoietic (45,706 cells) and stromal (14,577) cells. Of note, a myeloma-specific PC cluster was detected (Figures 4D and S5E). Cell-cycle analysis of myeloma cells showed that, as expected, most cells were in G1, with a minority in G2M and S phase, confirming ongoing cycling but consistent with the known low proliferation rate characteristic of myeloma cells (Figure 4H[i and ii]). These cells similarly expressed canonical myeloma markers including *BCL2, IRF4, JCHAIN, SLAMF7, TNFRSF13B*, and *TNFRSF17* (Figure 4H[iii]).

Differential abundance analysis (MiloR) revealed a significant enrichment in populations known to be altered in myeloma bone marrow when compared with controls (Figures 4I and 4J). MM-comBOs demonstrated an enrichment in lymphoid progenitors, T cell lineages, and mast cells/early mast cell progenitors in myeloma-engrafted organoids compared with un-engrafted controls, as expected given that these organoids had been engrafted with adult donor-derived lymphoid cells (Figure 4I). Similarly, an increase in early osteolineage cells, adipocytes, and endothelium were observed, with a bimodal distribution in osteoblasts/osteoblast progenitors and CD271+ MSCs, indicating significantly altered transcriptomes (Figure 4J).^40,42,43^

**Figure 5:**
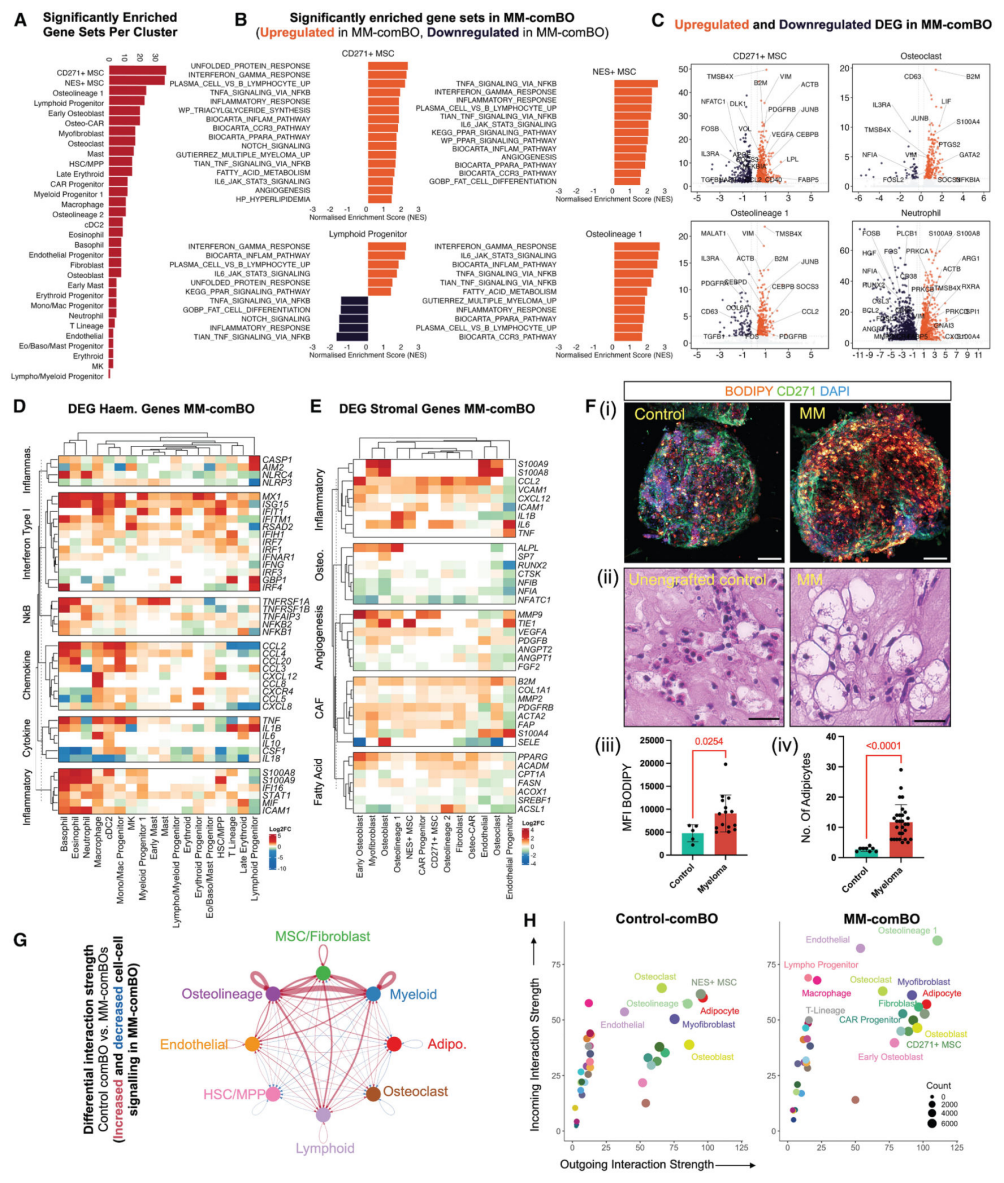
Multiple myeloma-engrafted comBOs capture hallmarks of complex disease pathology (A) Gene set enrichment analysis using hallmark gene sets and comparing myeloma-engrafted MM-comBO with control organoids reveals an extensive dysregulation, most evident in MSC, osteolineage cells, and lymphoid progenitors in MM-comBOs. (B) Representative GSEA plots highlight inflammatory response, fatty acid metabolism, and unfolded protein response genes as some of the major upregulated pathways in key clusters. (C) Volcano plots identifying key genes involved in inflammation, osteo/adipo-lineage fate, and fatty acid metabolism in key myeloma-associated cell types. (D and E) Heatmap of DEGs in specific pathways highlighted by GSEA in (D) hematopoietic and (E) stromal cells. (F) (i) Confocal imaging of BODIPY- and CD271-stained control and MM-comBOs demonstrates an increase in BODIPY+ cells (scale bar, 100 μm) and (ii) H&E staining of un-engrafted and MM-comBO confirmed a significant increase in adipocyte number (scale bar, 20 μm). Quantification across three donors vs. control confirmed a significant increase in (iii) BODIPY mean fluorescence intensity (MFI) (*p* = 0.0254) and in (iv) adipocyte number (*p* < 0.0001). Error bars represent standard deviation. (G) CellChat analysis identifies a dysregulation of cell-cell interactions across key clusters, identifying upregulated interactions in MM-comBO. (H) Analysis of net outgoing and ingoing signals reveals a substantial change in outgoing cell signals in MM-comBO, consistent with a remodeling of the organoid because of engraftment.

Gene set enrichment analysis (GSEA) comparing myeloma-engrafted comBO with controls identified significantly enriched hallmark gene sets across MM-comBOs, consistent with myeloma-induced remodeling of the comBOs (Figure 5A). A further analysis of the four cell types with the highest numbers of significantly enriched gene sets (CD271+ MSC, NES+ MSC, osteolineage 1, and lymphoid progenitors) highlighted inflammatory and metabolic gene sets (Figures 5B and S5F). Stromal cells (CD271+ MSC, NES+ MSC, and osteolineage 1) demonstrated tumor necrosis factor (TNF), IL-6, and interferon-mediated inflammatory responses, as well as an increase in fatty acid metabolism and PPARA signaling gene sets.^26,27,39–41,44^

Differentially expressed gene (DEG) analysis identified an up-regulation of master regulators of inflammation and differentiation in MM-comBOs (Figure 5C). Stromal activation markers (*ACTB, PDGFRB*, and *VIM*), angiogenic factors (*VEGFA*), adipogenic genes (*LPL, FABP5*, and *APOE*), and inflammatory genes (*NFKBIA* and *CCL2*) were significantly upregulated in CD271+ MSC (Figure 5C). Osteoclasts demonstrated an upregulation in markers of differentiation (*PTGS2, JUNB*, and *FOSL2*), activation and inflammation (*LIF, S100A4, NFKBIA*, and *B2M*) (Figure 5C).^39,42^ Early osteolineage cells (osteolineage 1) exhibited upregulation of *JUNB*, a stress- and inflammation-responsive transcription factor that has been associated with impaired osteoblast maturation under inflammatory conditions, and *SOCS3*, a canonical downstream effector of IL-6/STAT3 signaling that negatively regulates cytokine signaling and has been reported to inhibit osteoblast differentiation.^45,46^ *CCL2, CEBPB*, and *PDGFRB* upregulation similarly indicated an activated stromal compartment, with *CCL2* known to drive the recruitment of monocytes/macrophages that can facilitate osteoclast differentiation in myeloma (Figure 5C).^42^

Neutrophils have recently been reported as major drivers of myeloma inflammation.^27^ On myeloma engraftment, they upregulated inflammatory markers including *S100A8/A9*, alarmin axis proteins reported to impede immunotherapeutic efficacy in myeloma (Figure 5C).^47^

Differentially expressed inflammatory, cytokine, and chemo-kine genes were interrogated to identify an upregulation of interferon type I response genes, and nuclear factor κB (NF kB)-mediated TNF-α signaling was observed across the EBM cell arm, as well as in dendritic cells (cDC2) and monocyte/macrophage lineage cells (Figure 5D). A similar upregulation in chemokines (e.g., *CCL2, CCL3*, and *CCL4*) and alarmin axis (*S100A8* and *S100A9*)-mediated inflammation was also observed in neutrophils and EBM cells.^8,26,27,47^ Inflammasome-related *NLRC4* was upregulated in neutrophils and basophils, while *AIM2* and *CASP1* were upregulated in lymphoid progenitors in the MM-comBO.

In the stromal compartment, inflammatory genes (*S100A8, S100A9, CCL2, VCAM1, IL6*, and *TNF*), differential expression of osteolineage gene sets (e.g., the osteoblastic maturation genes *NFIA* and *NIFIB*), and an increase in angiogenesis genes (*TIE1, VEGFA*, and *ANGPT2*) were observed (Figure 5E). Similarly, genes associated with fibroblast activation in cancers (e.g., *FAP, S100A4, MMP2, COL1A1*, and *ACTA2*) were significantly upregulated, and dysregulation of fatty acid metabolism was observed (*PPARG, ACADM*, and *CPT1A*) (Figure 5E).

An increase in adiposity is a hallmark of myeloma-infiltrated bone marrow.^39,40^ To further investigate our transcriptomic data for evidence of an increase in lipid synthesis and fatty acid metabolism pathways (Figures 5B and 5E), we assessed MM-comBO derived from 3 patients for an increase in adipose cell number. We stained control and MM-comBOs for the lipid dye BODIPY (Figure 5F[i]) and assessed histological (H&E-stained) sections from MM-comBO (Figure 5F[ii]). Compared with control samples, we found a significant increase in BODIPY+ (Figure 5F[iii]) and a corresponding significant increase in adipocyte number (Figure 5F[iv]).

Together, GSEA and DEG analyses show that the engrafted malignant PCs extensively remodel the comBO microenvironment, recapitulating multilineage hallmarks of myeloma pathogenesis within a fully human system.

### Cell-cell interaction analysis reveals a druggable MIF-mediated inflammatory pathway

To investigate dysregulated cell-cell crosstalk in MM-comBOs, CellChat analysis was performed.^48,49^ The resulting interactome identified significantly more crosstalk in MM-comBOs, particularly in predicted ligand-receptor interactions across osteolineage cells and MSC/fibroblasts, consistent with DEG and GSEA data (Figure 5G).

Analysis of incoming and outgoing interaction strength, defined as total receptor and ligand expression, respectively, demonstrated a significantly altered distribution in MM-comBO samples when compared with controls. In MM-comBO (osteolineage 1, myofibroblast, adipocytes, endothelial cells, CAR progenitors, and osteoblasts) demonstrated relatively increased outgoing signals. Conversely, lymphoid progenitors, T-lineage cells, macrophages, and endothelial cells exhibited the most marked shift in incoming interaction strength (Figure 5H).

**Figure 6:**
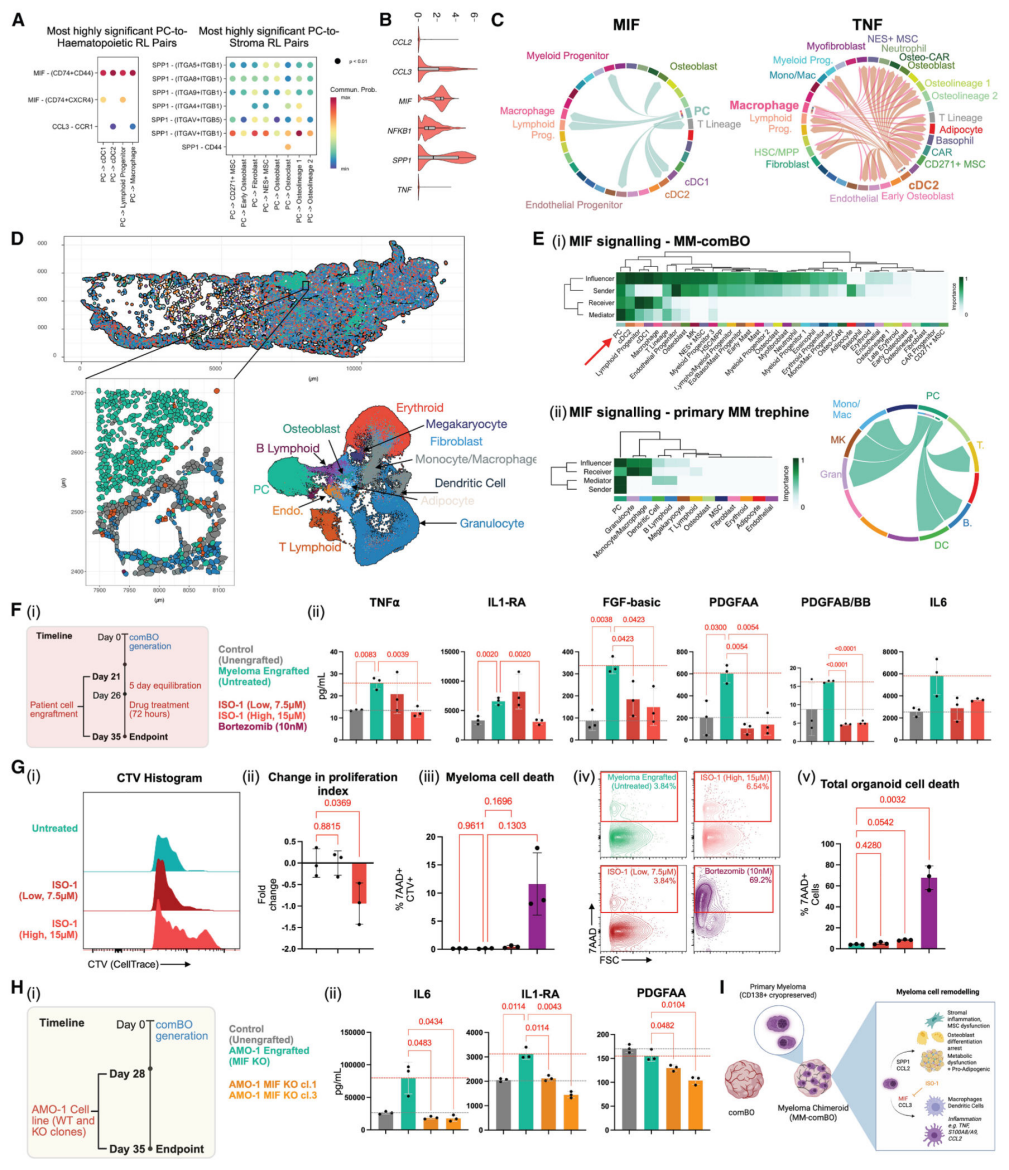
Cell-cell interaction analysis reveals mechanisms underpinning niche remodeling in myeloma potential therapeutic avenues (A) CellChat analysis reveals MIF, CCL3, and SPP1 as major ligand-receptor (LR) interactions originating from myeloma plasma cells (PCs), with MIF interactions highlighted as the most significant. (B) Violin plots confirm the expression of *MIF, SPP1*, and *CCL3*; however, while *TNF* is one of the major pathways disrupted in MM-engrafted comBOs, this is not expressed in patient cells despite its upregulation in myeloid cells (neutrophils, DCs, and Eo/Baso/Mast cells) in MM-comBOs. (C) Chord diagrams identify and confirm myeloma PC-derived MIF signaling to cDC2 and macrophage clusters, which in turn are responsible for the inflammatory TNF signals observed. (Chord diagrams depicting source of signaling pathway and color coded by cluster). (D) Xenium analysis of primary patient trephines captures a range of hematopoietic and stromal cell types. (E) (i) A clustered heatmap of centrality scores (CellChat) identifies PCs as the major source of MIF-mediated effects in the comBOs, confirmed by (ii) primary Xenium analysis of patient bone marrow trephine. Full details shown in Figure S6. (F) MIF inhibition was tested using patient engrafted MM-comBOs. Samples from 3 patients were engrafted for 5 days before treatment with ISO-1 (MIF inhibitor) at low (7.5 μM) and high (15 μM) doses for a further 9 days with dosing performed every 72 h. Un-engrafted (gray) controls were taken from un-engrafted samples from two separate differentiations (*n* = 2). For myeloma-engrafted samples, each individual data point represents supernatant pooled from 8 organoids engrafted with cells from a single patient. MIF inhibition (ISO-1 treatment) significantly mitigated the upregulation of TNFα, IL1Ra, FGF-basic, PDGF-AA, and PDGF-AB/BB. (Error bars are standard deviation. Significance determined with a Kruskal-Wallis test with an uncorrected Dunn’s test, alpha threshold and confidence level = 0.05). (G) (i) Representative CellTrace Violet (CTV) traces following MIF inhibition (ISO-1) (ii) with a significant (*p* = 0.0164) decrease in myeloma cell proliferation observed high dose of ISO-1 in 3 treated patient samples. (iii) No significant change in cell death was observed in myeloma cells (CTV^+^ cells) in ISO-1-treated samples, while a significant increase in cell death was observed in bortezomib-treated positive controls (*p* = 0.0164). (iv and v) Contour plots and quantification of total organoid cell death in treated samples show minimal cytotoxicity in MIF-treated samples, while significant cell death is observed in bortezomib-treated samples. (*n* = 3. Error bars are standard deviation. Significance determined with a Brown-Forythe and Welch ANOVA test with two-stage lineage step-up procedure of Benjamini, Krieger, and Yekutieli with individual variances computed for each comparison. Alpha threshold and confidence level = 0.05.) (H) (i) MIF knockout AMO-1 cell line clones were engrafted into comBOs at day 28 and assayed after 7 days of engraftment. Schematic created on Biorender.com. (ii) Luminex analysis of secreted IL6, IL1RA, and PDGF-AA confirmed a decrease in MIF KO when compared to wild-type (WT) AMO-1s engrafted into comBOs. (*n* = 3. Error bars are standard deviation. Significance determined with a Brown-Forythe and Welch ANOVA test with two-stage lineage step-up procedure of Benjamini, Krieger, and Yekutieli with individual variances computed for each comparison. Alpha threshold and confidence level = 0.05.) (l) Schematic showing differential interactions that drive elements of the MM-mediated pathological remodeling of comBOs, capturing hallmarks of disease and highlighting MIF inhibition as a route toward mitigating MM-mediated inflammation. Created on Biorender.com.

We next examined distinct crosstalk interactions between myeloma cells (PCs) and stromal or hematopoietic compartments within the MM-comBO (Figure 6A). *MIF*-(*CD74+CD44*), *MIF-(CD74+CXCR4*) and *CCL3*–*CCR1*-mediated interactions were the highest confidence pathways between MM cells and dendritic cells, lymphoid progenitors, and macrophages. In contrast, *SPP1*-mediated interactions were the strongest interacting partners between MM and stromal compartments. Expression of *MIF, SPP1*, and *CCL3* was evident in myeloma cells (Figure 6B), although *TNF* was not expressed, suggesting that the TNF-α signaling enriched in MM-comBOs was due to secondary effects, i.e., pro-inflammatory reprogramming of myeloid cells. DEG analysis identified myeloid cells (macrophage, cDC2, basophils, and eosinophils) as the major source of TNF-α in MM-comBO (Figure 5D).

Mapping the MIF signaling interactome confirmed that PC-derived MIF was received primarily by myeloid cells, including dendritic cells and macrophages (Figure 6C). Upregulated TNF signaling in MM-comBO was in turn primarily derived from macrophages and dendritic cells (cDC2) (Figure 6C).

These data implicated PC-derived MIF in myeloma-associated inflammation. We sought to confirm the relevance of these findings in clinical specimens. Xenium spatial transcriptomics using a custom panel was applied to bone marrow trephines from patients with newly diagnosed and relapsed disease (*n* = 3) (Table S2). Xenium data was annotated to identify PCs, hematopoietic, and Stromal cells (Figures 6D and S6A–S6E). CellChat network centrality analysis was performed on both primary trephine data (Xenium) and MM-comBOs. In both sample types, clustered heatmaps of critical senders (ligand-expressing cells), receivers (receptor-expressing cells), mediators (expression of both receptors and ligands), and influencers (combination of sending, receiving, and mediating roles) in MIF signaling identify PCs as the critical driver of MIF signaling networks (Figures 6E and S6A–S6C).

To functionally validate the role of MIF in myeloma-mediated inflammation, we designed inhibition experiments. MM-comBOs were generated using MM cells from 3 patients, and a MIF inhibitor (tautomerase binding ISO-1) was introduced at day 5 post engraftment.^50^ At day 14 post engraftment, supernatant was taken from treated samples and subject to Luminex analysis for markers of inflammation and metabolic dysfunction (Figure 6F[i]).

Inflammatory markers (TNF-α and IL1-RA) and stroma mediating factors ([fibroblast growth factor] FGF-basic, [platelet-derived growth factor] PDGF-AA, and PDGF-AB/BB) were upregulated in myeloma-engrafted organoids (Figure 6F[ii]). The MIF inhibitor ISO-1 significantly decreased this upregulation (Figure 6F[ii]), indicating an effective inhibition of MM-induced niche inflammation and stromal/endothelial interactions. High dose ISO-1 treatment induced a significant reduction in myeloma cell proliferation (Figure 6G), with no effect on either myeloma or total organoid viability (Figure 6G), suggesting a growth arrest effect rather than direct toxicity to myeloma cells. Interestingly, MM-comBOs treated in parallel with the protease inhibitor bortezomib (10 nM) demonstrated a high level of organoid and myeloma toxicity (Figure 6G), in keeping with the bone marrow toxicity seen clinically with this drug. These findings support MIF inhibition as a potential anti-myeloma strategy.

To validate these findings, we generated MIF CRISPR knockouts (KOs) in the myeloma cell line AMO-1 (Figures 6H[i] and S6F). Two clones with a validated protein level KO of MIF were selected and engrafted into comBOs before a Luminex-based assessment of major inflammatory markers. Significant reductions in IL6, IL1-RA, and PDGF-AA were observed upon MIF KO, confirming the role of MIF in stimulating myeloma-mediated inflammation (Figure 6H[ii]).

Together, our data report extensive remodeling of comBOs by myeloma cell engrafting, recapitulating hallmarks of disease (inflammation and transcriptional re-wiring of MSCs and osteolineage cells). These data highlight potential mechanisms by which myeloma PCs drive remodeling, including a MIF signaling axis which was validated in primary patient trephines and through inhibition assays (Figure 6I).

## Discussion

We present comBO, a multi-niche organoid system that integrates the osteoblastic, lymphopoietic, and myeloid compartments of human bone marrow. Unlike previous bone marrow organoid platforms,^22–25^ comBO captures both the myeloid microenvironment of the central marrow and the osteoblastic niche critical for lymphopoiesis, HSPC maintenance, and modeling hematopoietic diseases.^3,5,7,51^ By combining physiologically relevant oxygen levels with graded cytokine exposure and a 3D scaffold, comBO more faithfully recapitulates the structural and functional complexity of native human bone marrow.

Balancing complexity with scalability and reproducibility remains a major challenge in organoid research. The microgel-based approach used in comBO overcomes limitations of bulk hydrogels, enabling a single-step, cost-effective differentiation protocol that substantially reduces hands-on time and hydrogel usage, facilitating implementation in higher-throughput discovery and validation pipelines.

Serial transplantation into immunocompromised mice remains the gold standard for assessing HSC function.^52^ While recent organoid models capture aspects of hematopoiesis,^23–25^ serial transplantation in a fully human *in vitro* context remains rare. Frenz-Weissner et al. recently reported limited primary *in vivo* engraftment of CD34+ cells generated from BMO, with low-level human chimerism detected in a subset of transplanted mice.^23,25^ However, as comBO generates a broad range of hematopoietic and stromal cell types, the absolute number of HSC/HSPCs is relatively low, precluding conventional transplantation assays that require large cell numbers. To address this, we established a serial organoid re-seeding assay to assess whether the comBO niche could sustain CD34+ cells with lympho-myeloid potential over the long term in the absence of exogenous cytokines, and whether comBO-derived CD34+ cells retain lympho-myeloid output following serial passage. After three re-seeding cycles, both donor-derived and comBO-derived CD34+ cells preserved CD34 expression and continued to generate myeloid and lymphoid lineages. In contrast to small-molecule-based *in vitro* expansion systems such as SR1 or UM171,^52–54^ which maintain functional human HSCs with lympho-myeloid potential for up to 3 weeks,^55^ the comBO niche supports CD34+ cells with lymphomyeloid output for 6 weeks under growth factor-free conditions. While further work is required to delineate reconstitution dynamics and stem cell potential more precisely, this represents a substantial advance in modeling stem cell kinetics in a fully human *ex vivo* system.

Beyond myelopoiesis, comBO generates lymphoid progenitors and cells, including T cell and B cell progenitors. We validated the functional capacity of comBO-derived CD7+ T cell progenitors through an ATO system. Further work will further assess the functional and immunogenic potential of comBO-derived lymphoid populations and coupling comBO with primary lymphoid organoids to enable more complex immune modeling. We applied comBO to multiple myeloma as an exemplar bone marrow disease wherein malignant cells remodel the niche to support growth and modulate therapy response.^8,26,39,40,44,56–58^ Primary myeloma cells have been extremely difficult to culture *ex vivo*, mouse models poorly emulate the disease, and no existing culture platform captures the diverse stromal and hematopoietic elements contributing to disease progression, relapse, and the characteristic disruption of bone homeostasis, presenting a major bottleneck in myeloma research.^56^ comBO-engrafted primary myeloma cells survive and cycle, recapitulating complex interactions with the microenvironment and enabling the investigation of inflammatory mechanisms underlying disease progression and drug response. Targeting inflammation in myeloma holds therapeutic relevance in preventing the progression of precursor disease, bone loss, and enhancing responses to immunotherapy, making it a key area of priority research. Pro-inflammatory cyto-kines such as IL-6, TNF-α, IL-1B, CCL, IL1-RA, and PDGF-AA have been implicated in myeloma-associated bone disease by enhancing osteoclast activation and inhibiting osteoblast function.^8,26,27,59^ Our data implicate MIF in MM-mediated inflammation, a finding consistent with reports linking MIF to T cell exhaustion and resistance to proteasome inhibitors in MM.^60,61^ Using spatial transcriptomics from patient trephines and pharmacological inhibitors, we validated the role of MIF and demonstrated its potential as a therapeutic target. MIF inhibition significantly reduced inflammatory cytokine secretion in engrafted comBOs across three patients,^39^ and CRISPR-mediated MIF knockout in a myeloma cell line confirmed its causal role (Figure 6I).

These data establish comBO as a preclinical platform that both identifies and validates potential targets using patient material. More broadly, comBO enables systematic dissection of how stromal, vascular, and inflammatory niches regulate human hematopoiesis and disease progression—processes that have been difficult to study *ex vivo* in the absence of complex human models.^7,8^

In the absence of dynamic flow, the vasculature generated by comBO and other static organoid systems is not long-lived. We demonstrate proof of concept that dynamic fluid flow enhances vascular elaboration, and future work will assess its impact on vessel and stromal organization and maturation, hematopoiesis, and the feasibility of longer-term cultures to study clonal evolution and therapy resistance.

In summary, comBO represents a scalable and physiologically relevant human bone marrow organoid that models multiple niche compartments, bridging critical translational gaps and enabling mechanistic studies of hematopoiesis and blood disorders. comBO represents one of the most faithful human organoid models in terms of cell complexity and function, showcasing the potential and power of these systems for discovery and translational research. Beyond blood cancers, comBO can meet a major need for a scalable source of blood and immune cells in multi-organ modeling.^62^

### Limitations of the study

While we comprehensively characterize comBO using flow cytometry, imaging, and RNA sequencing, further work is needed to delineate the long-term stability of cellular composition, niche architecture, and Sustained hematopoiesis over extended culture periods. The use of multiple flow cytometry panels currently limits longitudinal quantification, and unified spectral panels alongside high-content imaging will be valuable to define population dynamics and spatial architecture. Further validation of stromal subsets, including adipocyte-like cells and transcriptionally distinct vascular niches, is required. Although comBO is reproducible across three hiPSC lines, broader validation across genetically and ethnically diverse lines will strengthen its reproducibility and generalizability.

The relatively low abundance of HSC-like cells currently precludes *in vivo* transplantation, and while our serial organoid reseeding assay demonstrates sustained CD34+ output over 6 weeks *ex vivo*, additional analyses will be needed to establish definitive HSC function. Similarly, further work is necessary to evaluate lymphoid functionality and to expand disease modeling across larger patient cohorts with longitudinal analyses of disease evolution. Last, incorporating dynamic perfusion systems, real-time imaging, and targeted modulation of predicted ligand-receptor pathways (e.g., CXCL12-CXCR4, NOTCH, and WNT) will provide deeper mechanistic and physiological insight into their functional roles in niche establishment.

### Resource Availability

#### Lead contact

Further information and requests for resources and reagents should be directed to the lead contact, Abdullah O. Khan (abdullah.khan@imm.ox.ac.uk).

#### Materials availability

Cell lines and other unique resources in this study are available upon reasonable request, with a completed materials transfer agreement.

## Star★Methods

### Key Resources Table

**Table:** 

REAGENT or RESOURCE	SOURCE	IDENTIFIER
Antibodies		
Anti-human CD45 APC-ef780	BioLegend	Cat#368516; RRID: AB_2566376
Anti-human CD34 BV650	BioLegend	Cat#343624; RRID: AB_2632725
Anti-human CD38 PE/Dazzle 594	BioLegend	Cat#303538; RRID: AB_2564105
Anti-human CD45RA BV570	BioLegend	Cat#304132; RRID: AB_2563813
Anti-human CD90 BV711	BD Biosciences	Cat#740786; RRID: AB_2740449
Anti-human CD123 PE-Cy7	eBioscience	Cat#25-1239-42; RRID: AB_1257136
Anti-human CD3 PerCP-Cy5.5	Biolegend	Cat#300430; RRID: AB_893299
Anti-human CD11b PerCP-Cy5.5	Biolegend	Cat#301328; RRID: AB_10933428
Anti-human CD14 PerCP-Cy5.5	Biolegend	Cat#301824; RRID: AB_893251
Anti-human CD19 PerCP-Cy5.5	Biolegend	Cat#302230; RRID: AB_2073119
Anti-human CD20 PerCP-Cy5.5	Biolegend	Cat#302326; RRID: AB_893283
Anti-human CD56 PerCP-Cy5.5	Biolegend	Cat#362506; RRID: AB_2563915
Anti-human CD235ab PerCP-Cy5.5	Biolegend	Cat#306614; RRID: AB_10683170
Anti-human CD11b AF700	BioLegend	Cat#301355; RRID: AB_2750074
Anti-human CD14 AF700	BioLegend	Cat#367113; RRID: AB_2566715
Anti-human CD117 (c-kit) BV711	BioLegend	Cat#313230; RRID: AB_2566217
Anti-human FceRIa APC	BioLegend	Cat#334612; RRID: AB_10578086
Anti-human CD41 FITC	BioLegend	Cat#303704; RRID: AB_314374
Anti-human CD42a PE	BD Biosciences	Cat#558819; RRID: AB_397130
Anti-human CD71 BV510	BD Biosciences	Cat#744926; RRID: AB_2742586
Anti-human CD235ab PE-Cy7	BioLegend	Cat#306620; RRID: AB_2716164
Anti-human CD10 PerCP-Cy5.5	Biolegend	Cat#312216; RRID: AB_10642819
Anti-human CD5 PE	Biolegend	Cat#300608; RRID: AB_314094
Anti-human CD7 PE-Cy5	Biolegend	Cat#343110; RRID: AB_2075096
Anti-human CD19 APC	Biolegend	Cat#302212; RRID: AB_314242
Anti-human CD56 BV605	Biolegend	Cat#318334; RRID: AB_2561912
Anti-human CD271 BUV395	BD Biosciences	Cat#743362; RRID: AB_2741453
Anti-human CD45 FITC	BioLegend	Cat#368508; RRID: AB_2566368
Anti-human CD31 APC-Cy7	BioLegend	Cat#303120; RRID: AB_10640734
Anti-human CD144 PerCP-Cy5.5	BioLegend	Cat#348510; RRID: AB_2562928
Anti-human CD90 PE-Cy5	BioLegend	Cat#328112; RRID: AB_893432
Anti-human ALP BUV737	BD Biosciences	Cat#748692; RRID: AB_2873096
Anti-human LEPR AF647	BD Biosciences	Cat#564376; RRID: AB_2738777
Anti-human CD3 BUV395	BD Biosciences	Cat#563546; RRID: AB_2744387
Anti-human CD4 BUV805	BD Biosciences	Cat#612887; RRID: AB_2870176
Anti-human CD7 FITC	Biolegend	Cat#343103; RRID: AB_1659217
Anti-human CD8 PE-Cy7	Biolegend	Cat#344750; RRID: AB_2687201
Anti-human TCR α/ β Pacific Blue	Biolegend	Cat#306715; RRID: AB_1953256
Anti-human CD138 BV711	BD Biosciences	Cat#563184; RRID: AB_2738054
Anti-human CD56 FITC	Biolegend	Cat#362546; RRID: AB_2565964
Anti-human CD31	Abcam	Cat#ab28364; RRID: AB_726362
Anti-human CD34	BioLegend	Cat#343502; RRID: AB_1731898
Anti-UEA1 biotinylated	Vector Laboratories	Cat#B-1065; RRID: AB_2336766
Anti-CD271	ThermoFisher Scientific	Cat#MA5-31968; RRID: AB_2809262
Anti-CD3	ThermoFisher Scientific	Cat#MA5-12577; RRID: AB_10979571
Anti-CD10	ThermoFisher Scientific	Cat#MA5-27893; RRID: AB_2744947
Anti-osteopontin	ThermoFisher Scientific	Cat#MA5-17180; RRID: AB_2538651
Goat anti-mouse Alexa Fluor 488	ThermoFisher Scientific	Cat#A-11001; RRID: AB_2534069
Goat anti-rabbit Alexa Fluor 647	ThermoFisher Scientific	Cat#A-21245; RRID: AB_2535813
Streptavidin Alexa Fluor 568 conjugate	ThermoFisher Scientific	Cat#S-11226; RRID: AB_2315774
Anti-MIF	Abcam	Cat#ab65869; RRID: AB_2144068
Biological samples		
Healthy donor CD34+ cells	University of Oxford INForMed Study	IRAS: 199833; REC 16/LO/1376
Multiple myeloma patient cells	Oxford Radcliffe Biobank	REC 19/SC/0173
Multiple myeloma patient bone marrow trephine	Oxford Radcliffe Biobank	REC 19/SC/0173
Chemicals, peptides, and recombinant proteins		
Geltrex	ThermoFisher Scientific	Cat#A1569601
StemFlex	ThermoFisher Scientific	Cat#A3349401
ReLeSR	Stem Cell Technologies	Cat#100-0483
RevitaCell	ThermoFisher Scientific	Cat#A2644501
STEMdiff APEL2 medium	Stem Cell Technologies	Cat#05275
CHIR99021	Stem Cell Technologies	Cat#72054
BMP4	PeproTech	Cat#120-05
FGF2	PeproTech	Cat#100-18B
VEGFA	PeproTech	Cat#100-20
SCF	PeproTech	Cat#300-07
Flt3-L	PeproTech	Cat#300-19
VEGFC	PeproTech	Cat#100-20CD
TPO	PeproTech	Cat#300-18
EPO	PeproTech	Cat#100-64
IL3	PeproTech	Cat#200-03
IL6	PeproTech	Cat#200-06
IL7	PeproTech	Cat#200-07
mCSF	PeproTech	Cat#300-25
Matrigel Growth Factor Reduced (GFR) Basement Membrane Matrix	Corning	Cat#354230
StemPro-34 SFM	ThermoFisher Scientific	Cat#10639011
KnockOut Serum	ThermoFisher Scientific	Cat#10828028
Heparin	Panpharma	Cat#B01AB01
Fibrinogen	Merck	Cat#F8630-5G
VitroCol	Advanced Biomatrix	Cat #5007
Thrombin	Merck	Cat#605157
VitroCol Type I Collagen Lyophilized Powder Human	Advanced Biomatrix	Cat#5008
Type IV Collagen Lyophilized Powder Human	Advanced Biomatrix	Cat#5022
Collagenase D	Roche	Cat#11088866001
Hanks Balanced Salt Solution (HBSS)	Sigma	Cat#H9394
DNase I	Roche	Cat#10104159001
MethoCult H4435 Enriched	Stem Cell Technologies	Cat#04435
IMDM (Iscove’s Modified Dulbecco’s Medium)	Gibco	Cat#12440053
Chemically Defined Lipids	ThermoFisher Scientific	Cat#11905031
Human albumin	Sigma	Cat#A5843-5G
Insulin-free B27	Gibco	Cat#A1895601
ITSE (Insulin-Transferrin-Selenium- Ethanolamine (ITS -X))	Gibco	Cat#51500056
alpha-MEM media	Gibco	Cat#12571063
HEPES	Gibco	Cat#15630080
b-mercaptoethanol	Gibco	Cat#21985023
penicillin-streptomycin-glutamine	Gibco	Cat#10378016
RPMI-1640	Gibco	Cat#11875093
L-ascorbic acid 2-phosphate	Sigma	Cat#49752-10G
Penicillin-Streptomycin	Gibco	Cat#15140122
GlutaMAX	Gibco	Cat#35050061
ISO-1	MedChemExpress	Cat#HY-16692
Bortezomib	MedChemExpress	Cat#HY-10227
CellTrace Violet Cell Proliferation Kit	ThermoFisher Scientific	Cat#C34557
DAPI	Life Technologies	Cat#D3571
7AAD	Cayman Chemical	Cat#11397
Propidium iodide	Sigma	Cat#P4864
Critical commercial assays		
Chromium Next-GEM 3’ kits v3.1	10x Genomics	Cat#1000269
10x Genomics 3’ HighThroughput kit v3.1	10x Genomics	Cat#1000370
Human XL Cytokine Luminex Kit performance Assay	R&D Systems	Cat#FCSTM18B
Xenium v1 workflow	10x Genomics	Protocol CG000749; revision A
Deposited data		
Raw and processed scRNA-seq	This paper	GEO: GSE287648
scRNA-seq of bone marrow organoids	Khan et al.^23^	GEO: GSE196684
scRNA-seq of bone marrow organoids	Frenz-Weissner et al.^25^	GEO: GSE249005
Single cell adult bone marrow atlas	Bandyopadhyay et al.^2^	GEO: GSE253355
Single cell fetal bone marrow atlas	Jardine et al.^33^	GEO: GSE166895
Experimental models: Cell lines		
Gibco hiPSC	ThermoFisher Scientific	Cat#A18945; RRID: CVCL_RM92
SCTi003-A hiPSC	Stem Cell Technologies	Cat#200-0511; RRID: CVCL_C1W7
KOLF2.1J hiPSC	The Jackson Laboratory	Cat#JHIPSC1000; RRID: CVCL_B5P3
MCND-TENS2-mScarlet3 hiPSC	National Institutes of Health (NIH) iPS Cell Core Facility	N/A
MS5-hDLL4 stromal cells	Anindita Roy Lab	N/A
AMO1 cell line	DSMZ	RRID: CVCL_1806
Oligonucleotides		
gRNA for MIF: CACAGCATCGGCAAGATCGG[CGG]	This paper	N/A
Recombinant DNA		
Plasmid: pX458-mRuby	Hertzog et al.^63^	Addgene, Cat#110164
Software and algorithms		
Graphpad Prism (v.10.3.1)	GraphPad	https://www.graphpad.com/
Cell Ranger (v.7.0.0)	10X Genomics	https://www.10xgenomics.com/support/software/cell-ranger
CellBender (v.0.3.0)	Broad Institute	https://github.com/broadinstitute/CellBender
Souporcell (v.3.0.0)	GitHub repository wheaton5	https://github.com/wheaton5/souporcell
Seurat (v.5.1.0)	R package	https://satijalab.org/seurat/
CellChat (v.1.6.1)	R package	https://github.com/sqjin/CellChat
miloR package (v.2.0)	R package	https://bioconductor.org/packages/release/bioc/html/miloR.html
Harmony (v.1.2.0)	R package	https://cran.r-project.org/package=harmony
scCustomise package (v.2.1.2)	R package	https://samuel-marsh.github.io/scCustomize/
FlowJo v.10.10	BD	https://www.fiowjo.com/fiowjo10/overview
Other		
Ultra-low attachment plates 6-well	Corning	Cat#3471
Ultra-low attachment plates 96-well U- Bottomed	Corning	Cat#7007
Electroporation cuvette	Scientific Laboratory Supplies	Cat#FBR-204

### Experimental Model And Study Participant Details

#### Cell lines

Human induced pluripotent stem cells were maintained on Geltrex-coated plates in StemFlex maintenance medium. Four hiPSC lines were used in this study, Gibco hiPSC, SCTi003-A, KOLF2.1J, and MCND-TENS2-mScarlet3. Cells were maintained during routine passaging in a 37°C incubator at 5% CO_2,_ with routine clump passaging performed using ReLeSR. Cells were mycoplasma tested at 1-month intervals, with karyotyping performed on purchase/import.

MS5-hDLL4 stromal cells were maintained as adherent cultures in alpha-MEM media supplemented with 10% fetal bovine serum (FBS), 55 μM b-mercaptoethanol, 1% HEPES, and 1% penicillin-streptomycin-glutamine. AMO1 multiple myeloma cells were cultured in RPMI-1640 media supplemented with 10% FBS and 1% penicillin-streptomycin at 37°C and 5% CO_2_.

#### Primary cells

Healthy donor CD34+ cells were obtained through the INForMed Study, University of Oxford (IRAS: 199833; REC 16/LO/1376). Written informed consent was received for all the participants for the donation of human samples. Primary cells used for myeloma comBO engraftment, and bone marrow trephine samples used in spatial transcriptomic experiments, were all obtained from the Oxford Rad-cliffe Biobank, REC approval reference 19/SC/0173 (see details in Table S2).

### Method Details

#### Organoid differentiation

A full list of all media compositions and cytokine cocktails is provided in Figure S1A. Briefly, the comBO differentiation protocol involves a clump dissociation (day -1) in StemFlex supplemented with RevitaCell and subsequent culture in ultra-low attachment 6-well plates. On day 0, the resulting aggregates are collected by centrifugation (100xG 3 minutes, room temperature). Media is then aspirated before cells were resuspended in APEL2 supplemented with BMP4, FGF2, VEGFA and CHIR99021 and cultured in 5% oxygen. Note that cells are maintained in 5% O_2_ for a total of 20 days under the comBO protocol, and as such care must be taken when performing media changes to avoid a prolonged exposure to atmospheric oxygen. On day 3, embryoid bodies are collected by gravitation and media is then aspirated before resuspension in APEL2 supplemented with VEGFA, BMP4, FGF2, hSCF, FLT3L. On day 5, cells were either prepared for embedding in bulk hydrogels or in a granular microgel. Bulk hydrogel samples were prepared as follows: First, a 12-well tissue culture plate was washed with 1mL phosphate buffered saline (PBS). For each well required, 500 μL of hydrogel is prepared.

The first (base layer) is a fibrin-collagen type-1 mixed hydrogel generated by mixing fibrinogen and VitroCol, with a hydrogel diluent, thrombin, and 1M NaOH on ice to a final concentration of 1.25 mg/mL and 2 mg/mL respectively. The hydrogel diluent described by Olijnik et al.^24^ is used to dilute the hydrogel mixture. Once prepared and added to a 12-well tissue culture plate, the first layer of hydrogel is incubated at 37°C for 90 minutes. Once the base layer is polymerised, cell aggregates are collected by gravitation and resuspended in the second hydrogel layer. Approximately 40 aggregates are cultured in each well. The second layer is comprised of fibrinogen (1.25 mg/mL), thrombin (2 U/mL), VitroCol (2 mg/mL), and collagens type:I-IV (3 mg/mL, 1 mg/mL respectively, diluted with hydrogel diluent, and neutralised with NaOH).

Once mixed, the cell-containing second hydrogel layer should be added dropwise to the first gel layer before incubation at 37°C, 5% CO_2_, for 90 minutes. Once fully polymerised, prepare 1 mL of APEL2 for each well of a 12-well plate as described in Figure S1A and add dropwise. On day 7, 1 mL of media is added as described in Figure S1A. From day 10 onwards, media changes are performed at a ratio of 50:50 conditioned to fresh medium. On day 12, sprouted aggregates are collected using a pasteur pipette and sub-cultured in 96-well ULA plates as individual organoids, with media changes as described in Figure S1A.

For granular microgels, a volume of layer 2 hydrogel solution is prepared on day 5 and transferred into a sealed sterile syringe and allowed to polymerise for 90 minutes before fragmentation by passing through a series of sterile needles (18G, 21G, 23G, 25G, 27G).

The resulting fragmented gel is resuspended in a volume of APEL2 day 5 medium. A total of 450 μL of gel is prepared per 96-well plate required, diluted to a final volume of 5 mL in day 5 medium. A total of 120 cell aggregates are re-suspended in a 5 mL volume to ensure a complete 96-well plate. The final 5 mL volume, comprised of cell aggregates, growth factors, and fragmented microgel, is distributed into a 96-well ULA using a reagent reservoir, with a volume of 50 μL per well dispensed. On day 7, a further 50 μL of complete media is added per well. From day 10 onwards, partial (50:50) media changes are performed using a multi-channel pipette. Note that users should take care not to accidentally aspirate organoids at early stages, a schematic detailing this protocol is shown in Figure S4B.

#### Flow cytometry

Organoids were collected by gravitation into tubes and washed twice with PBS prior to dissociation. Dissociation was performed using collagenase D (dissolved in HBSS) at a final concentration of 2.5 mg/mL and supplemented with 1% FBS and 1000 U/mL DNase I in IMDM medium, as described by Schreurs et al.^64^ Typically, 1 mL of this dissociation solution could be used for dissociating 20 organoids. Organoids were incubated at 37°C for 15-30 minutes, with pipette trituration performed every 5-8 minutes, until a single-cell suspension was obtained. Digestion was stopped by adding a stopping buffer consisting of PBS, 10% FBS, and 20 mM EDTA. Cells were then centrifuged at 300xG for 5 minutes, and the cell pellets were resuspended in flow cytometry buffer (PBS + 10% FBS + 2 mM EDTA) for antibody staining. Following staining, samples were washed with flow cytometry buffer and analysed using a BD LSR Fortessa X20 flow cytometer. Single-stained controls and fluorescence-minus-one controls were used for the panel setup with antibodies listed in Table S1. Flow cytometry data were analysed in FlowJo v.10.10 software.

#### Imaging

Organoids used for imaging were fixed with 4% paraformaldehyde on a roller at room temperature for 1h, then stored in PBS at 4°C for further processing. Imaging of fluorescently labelled organoids (antibody details in key resources table) was carried out using a Zeiss LSM 880 Axio Observer Z1 confocal microscope, running Zen 2.3 SP1 FP3 (Black), with a Plan-Apochromat 10x/0.45 or a C-ACHROPLAN 32x/0,85 W Korr VIS-IR objective at 0.6 x zoom, taking Z-stacks of various height and scaling [1.384 x1.384x8μm @10x and 0.216x0.216x3μm @32x], with a pixel dwell time of 0.7 μs and 4 x line average. Fluorescent labelling and subsequent clearing with ethyl cinnamate was performed as described by Olijnik et al.^24^ Histology imaging was performed using an EVOS M5000 (Thermo Scientific). Histology imaging was white balanced using a custom ImageJ macro. Fluoerscent images were pre-processed using background subtraction (rolling ball) before LUT adjustment for visualisation purposes. Typically, 3D projections were used for Z-stack images.

#### Single cell RNA sequencing

For all single-cell RNA sequencing experiments, cells were from a total of 4 independent differentiations. For day 20 comBO sequencing, these were cryopreserved and thawed before live cell sorting and processing with the Chromium Next-GEM 3’ kits v3.1, 4 rxns using the Dual Index 3’ protocol (CG000315). For day 35 comBO, healthy donor and MM patient-engrafted samples were prepared fresh on day 35 of the experiment by collagenase D dissociation and live cell sorting before loading on the 10x Genomics 3’ HighThroughput kit v3.1. Two unengrafted controls were used for this experiment, each from independent differentiations. One was cryopreserved and thawed prior to live cell sorting, the other was prepared in parallel to engrafted samples.

#### Analysis of single cell RNA sequencing

Single cell data was processed using the CellRanger pipeline (v.7.0.0), post-processed using CellBender (v.0.3.0),^65^ Souporcell (v.3.0.0)^66^ and Seurat (v.5.1.0).^67,68^ Predicted ligand receptor (LR) analysis was performed using CellChat (v.1.6.1).^48,49^ Differential abundances were computed with miloR package (v.2.0),^69^ integration performed using Harmony (v.1.2.0)^70^ and plotting performed using the scCustomise package (v.2.1.2). All raw data and processed data (R objects) are available to review on GEO accession GSE287648: https://www.ncbi.nlm.nih.gov/geo/query/acc.cgi?acc=GSE287648. All analysis scripts are available via GitHub (https://github.com/khanaswimm/comBO.v1). In summary, all CellRanger-aligned cells were processed for ambient RNA removal via CellBender, patient demultiplexed via Souporcell and imported as Seurat objects. Quality control filtering was performed on RNA features, counts, and percentage mitochondrial genes before normalization via SCTransformation (genes regressed were mitochondrial, ribosomal,and cell cycle genes). Typically, QC standards were: nFeature_RNA (min. 500, max 10,000), nCount_RNA (min. 1000, max. 60,000), and percentage mitochondrial genes below 15%. This would vary depending on data set, but is fully documented in the relevant GitHub

Data was then integrated with the Harmony package to form batch corrected seurat objects. (integration co-variance was sample) Based on the expression of canonical haematopoietic and stromal genes, objects were divided into haematopoietic and stromal objects for cluster analysis.

LR CellChat analysis was performed on all cells, merging the separated haematopoietic and stromal compartments. To highlight broad changes in LR interactions CellChat analysis was first performed on merged data from all of the organoid studies (either mBMO and d20 comBO, or control and MM-comBO data). Further analysis focussed on identifying key condition-specific LR pairs and changes on either d20_comBO data or MM-comBO data.

Lineage tracing analysis was performed on the hemopoietic compartment to validate the known HSC/MPP differentiation outputs observed experimentally. For this, we implemented Palantir (v1.4.1), CellRank (v2.0.7) and Moscot (v0.4.3) methodologies. Palantir models cell fate probabilities via markov chain to align differentiating cells along a pseudotime continous measurement. This is done by defining a root HSC/MPP cell and cells from known terminal differentiated states (e.g. erythroid, B cells). Pseudotime and differentiation probability towards terminal states are then used to align cells across the expected lineages. This visualises all directions of differentiation at once. The palantir pseudotime was inputted into the CellRank pseudotime kernel function. This enables visualisation on a k-nn graph while correcting differentiation directionality by down-weighting edges where the pseudotime directions points towards the origin. To validate the inferred trajectories, we deployed Moscot, a method which makes use of both gene expression and temporal dimension of the experimental setting. This computed cell transition probabilities of all cellular types from time point d21 to become a different cellular type in time point d35. Leveraging these cell transition matrices, the algorithm inferred parental cells of terminally differentiated states.

Differential Gene Expression and Gene Set Enrichment analysis were performed using Seurat’s built in find markers function and the fGSEA package. Where a cluster was only present in one of the data sets, this cluster would be removed to facilitate comparative analysis. Typically, an FDR of 0.20 was used with a logfc threshold based on the data in question (average 0.25). GSEA analysis was performed using a hallmark gene set which included bone marrow and inflammatory gene sets (uploaded in relevant Github). GSEA and DEG were performed by comparing like-for-like clusters in control (un-engrafted and HD engrafted) vs. MM engrafted organoids. Differential Abundance (DA) analysis was performed comparing un-engrafted controls vs. MM-comBO. Finally,

#### Methocult cultures

Live CD45+CD34+CD144-cells were sorted from day 21 dissociated comBOs on a FACSAria Fusion sorter (Becton Dickinson). Cells were plated in methylcellulose H4435 Enriched. After 14 days, colonies derived from erythroid (BFU-E, burst forming unit-erythroid) and granulo-monocytic (CFU-GM, colony forming unit granulocyte macrophage) progenitors were counted.

#### Serial organoid re-seeding assay

CD34+lineage-HSPCs derived from healthy donors (one cord blood, two peripheral blood) were sorted into comBOs at day 28 of organoid differentiation at a density of 100 cells per organoid. ComBOs were generated from the fluorescent hiPSC line MCND-TENS2-mScarlet3, created via CRISPR/Cas9-mediated knock-in of mScarlet3 at the AAVS1 safe harbour locus in the parental line MCND-TENS2 (registered at https://hpscreg.eu/cell-line/RTIBDi001-A). The knock-in was performed by the iPS Cell Core Facility at the National Institutes of Health (NIH), USA. Engrafted organoids were cultured at 20% O_2_ in growth factor-free media (IMDM, 2% C/D Lipids, human albumin (2.5mg/500mL), Insulin-free B27, ITSE, 1ng/mL of the liver derived factor TPO essential for stem cell function). At two weeks post-seeding, comBOs were dissociated and mScarlet-negative donor CD34+ cells were re-seeded into fresh day 28 comBOs at 100 cells per organoid for secondary re-seeding. Cultures were again maintained under cytokine-free conditions. After an additional two weeks, comBOs were dissociated and donor-derived CD34+ cells were re-seeded a third time at the same density (tertiary re-seeding). Two weeks following the tertiary re-seed, engrafted organoids were dissociated and analysed.

For comBO-derived CD34+ cell serial re-seeding, CD34+ cells were sorted from day 21 mScarlet+ hiPSCs-derived comBOs and engrafted into non-fluorescent comBOs. At each re-seed stage, mScarlet+CD34+ cells were sorted and re-seeded at 100 cells per organoid. After three sequential serial re-seeds under cytokine-free conditions, organoids were dissociated for analysis.

#### Artificial thymic organoid assay

150,000 MS5-hDLL4 stromal cells were co-aggregated with 2500 comBO-derived CD7+ cells, and plated onto 0.4 μm Millicell transwell inserts positioned in 6-well plates containing 1 mL of RPMI-1640 media supplemented with 4% B27, 30 μM L-ascorbic acid 2-phosphate, 1% penicillin/streptomycin, 1% GlutaMAX, 5 ng/mL SCF and Flt3L, and 2.5 ng/mL IL-7.^71^ Media was changed every 3-4 days. ATOs were harvested from transwell inserts, dissociated with trypsin for 2 minutes, and Stained for flow cytometry analysis.

#### Inhibitor experiments

Inhibitor experiments were performed by engrafting primary myeloma samples on day 21 of comBO differentiation. Cells from three myeloma patients were seeded at 20,000 cells per organoid and cultured for a further total of 14 days. Inhibitors were added at day 5 post-engraftment once cells had engrafted and recovered from thaw and engraftment. All inhibitors were added in a 50 μL volume at 2x concentration (with 50 μL being the volume within each organoid well prior to treatment). The final volume per well was 100 μL, and concentrations are listed as final concentration values. ISO-1 (MIF inhibitor) was used at 7.5 μM (low dose) and 15 μM (high dose), Bortezomib (10nM) with re-dosing (re-feeding) every 72 hours. At day 9 after treatment (14 days post-engraftment), supernatant from 8 organoid wells under the same condition was pooled and subject to Luminex cytokine assays.

#### CRISPR KO experiments

To generate MIF CRISPR knockout AMO1 cells, 5 million AMO1 cells were cultured in a 4 mm long electroporation cuvette with 500 uL media (10% FBS RPMI, antibiotic-free) and 5 μg plasmid (pX458-mRuby backbone ligated in gRNA sequence for MIF; CACAG-CATCGGCAAGATCGG[**CGG**]^63^). Cells were electroporated at 330 mV for 10 ms using EPI 2500 Electroporations-Impulsgenerator 0-2500 V (Fischer) and incubated at RT for 5 min. Cells were then transferred 5 mL media (20% FBS RPMI, antibiotic-free) in a 6-well plate. After 24h, cells were pelleted and resuspended in 0.5 mL FACS buffer (10% FBS, 2 mM EDTA in PBS) and single cell sorted using the BD FACSAria Fusion sorter. Single cell derived colonies were selected using MIF antibody (ab65869) for downstream assays.

#### Luminex multiplex analysis

For Luminex analysis, supernatant from 8 organoids per treatment condition and patient were pooled and spin at 500xG for 5 minutes before being placed on dry ice and subsequently stored at -80°C. Protein expression was measured using the Human XL Cytokine Luminex Kit performance Assay. Analysis was restricted to factors upregulated in engrafted samples when compared to un-engrafted controls.

#### Xenium spatial transcriptomics

Bone marrow trephine specimens were fixed in formalin, decalcified and then paraffin embedded. Specimen blocks were sectioned and mounted on Xenium slides, then underwent deparaffinisation and decrosslinking according to the manufacturer’s protocol. Targeted probes designed for a custom 480-parameter panel were applied along with cell segmentation staining according to the Xenium v1 workflow. Data were then acquired with the Xenium Analyzer. Downstream analysis was performed in R, using Seurat and CellChat.

### Quantification and Statistical Analysis

Statistical analyses of inhibitor, CRISPR, and flow cytometry data was performed using GraphPad PRISM 10 (v.10.3.1). Data are presented as mean ± SD. Details of specific statistical tests and the exact value of *n* for each experiment are listed in the figure legends of relevant experiments. The p values used were *p < 0.05, **p < 0.01, ***p < 0.001, ****p < 0.0001.

### Additional Resources

For troubleshooting and guidance on establishing bone marrow organoid protocols, we would recommend joining our online forum, where frequently asked questions and experimental details are discussed: https://groups.google.com/g/morerganoids.

## Data Availability

All raw data and processed data (fully annotated R objects) are available to review on GEO accession GSE: 287648. No new code was generated for this manuscript, and all analysis scripts are available via GitHub (https://github.com/khanaswimm/comBO.v1).

## In brief

Shen et al. report comBO, a combined human bone and bone marrow model that integrates osteolineage, vascular, stromal, and hematopoietic compartments in a single hiPSC-derived organoid system. comBO supports primary patient cells for preclinical disease modeling, identifying and validating MIF as a targetable inflammatory axis in myeloma-comBO chimeroids.

## Highlights

- comBO models stromal, vascular, and osteoblastic niches in a single organoid
- comBO supports sustained lymphoid and myeloid output in a fully human *ex vivo* system
- Primary myeloma cells proliferate and remodel the tumor microenvironment in comBO
- Patient chimeroids identify MIF as a targetable driver of inflammation in myeloma
